# Primaquine radical cure of *Plasmodium vivax*: a critical review of the literature

**DOI:** 10.1186/1475-2875-11-280

**Published:** 2012-08-17

**Authors:** George K John, Nicholas M Douglas, Lorenz von Seidlein, Francois Nosten, J Kevin Baird, Nicholas J White, Ric N Price

**Affiliations:** 1 Centre for Tropical Medicine, Nuffield Department of Clinical Medicine, University of Oxford, Oxford, UK; 2 Global Health Division, Menzies School of Health Research, Charles Darwin University, PO Box 41096, Casuarina, Darwin, NT 0811, Australia; 3 Vivax Working Group, Asia-Pacific Malaria Elimination Network, Menzies School of Health Research, Darwin, NT, 0811, Australia; 4 Shoklo Malaria Research Unit, Mae Sot, Tak, 63110, Thailand; 5 Mahidol-Oxford Tropical Medicine Research Unit, Bangkok, 10400, Thailand; 6 Eijkman-Oxford Clinical Research Unit, Jalan Diponegoro No. 69, Jakarta, 10430, Indonesia; 7 WorldWide Anti-malarial Resistance Network (WWARN), Centre for Tropical Medicine, Nuffield Department of Clinical Medicine, University of Oxford, Churchill Hospital, Old Road, Oxford, UK

## Abstract

**Background:**

Primaquine has been the only widely available hypnozoitocidal anti-malarial drug for half a century. Despite this its clinical efficacy is poorly characterized resulting in a lack of consensus over the optimal regimen for the radical cure of *Plasmodium vivax*.

**Methods:**

Published studies since 1950 of the use of primaquine regimens for preventing *P. vivax* relapse were reviewed. Data were extracted systematically from available papers. Primaquine regimens were categorized according to the total dose administered: very low (≤2.5 mg/kg), low (>2.5 mg/kg- < 5.0 mg/kg) and high (≥ 5.0 mg/kg). The risk of recurrent infection were summarized across geographical regions and the odds ratios between treatment regimens calculated after stratifying by total treatment dose and duration of study follow up.

**Results:**

Data could be retrieved from 87 clinical trials presenting data in 59,735 patients enrolled into 156 treatment arms, conducted in 20 countries. There was marked heterogeneity in study design, particularly primaquine dosing and duration of follow up. The median rate of recurrence following very low dose of primaquine (n = 44) was 25% (range 0-90%) at 4–6 months, compared to 6.7 % (range 0-59%) following low dose primaquine (n = 82). High dose primaquine regimens were assessed in 28 treatment arms, and were associated with a median recurrence rate of 0% (Range: 0-15%) at one month. In 18 studies with control arms, the effectiveness of a very low dose primaquine regimen was no different from patients who did not receive primaquine (OR = 0.60, 95%CI 0.33-1.09, p = 0.09), whereas for the low dose regimens a significant difference was reported in 50% (6/12) of studies (overall OR = 0.14, 95%CI: 0.06-0.35, p < 0.001). Two studies enrolling 171 patients demonstrated high effectiveness of high dose primaquine compared to a control arm (OR = 0.03 (95%CI: 0.01-0.13); p < 0.0001).

**Conclusions:**

Low dose regimens retain adequate efficacy in some areas, but this is not uniform. The efficacy and safety of pragmatic high dose primaquine regimens needs to be assessed in a range of endemic and geographical locations. Such studies will require a prolonged period of follow up and comparison with control arms to account for confounding factors.

## Background

Over 40% of the world’s population is at risk of infection with *Plasmodium vivax*, with an annual global burden estimated to be between 71 to 391 million clinical cases [1-3]. Vivax malaria causes significant morbidity and associated mortality, much of which is attributable to the chronic relapsing nature of the infection. As malaria control efforts intensify in malarious areas co-endemic for *P. vivax* and *Plasmodium falciparum*, the relative proportion of *P. vivax* tends to rise when compared to that of *P. falciparum*, reflecting the greater challenge for the control and elimination of this parasite. This can be explained by the ability *P. vivax* to develop hypnozoite stages, which can lie dormant for months or even years. The number and frequency of *P. vivax* relapses vary with a number of factors including the geographical region where the infection is acquired, host immunity and the sporozoite load at the time of infection. The geographical differences in relapse patterns have been attributed to the existence of different strains of *P. vivax* which vary in the proportion of sporozoites that are committed to dormancy, the duration of dormancy and the propensity of dormant stages to re-awaken [4]. The frequent relapse phenotype is epitomized by the Chesson strain isolated originally from the island of New Guinea and thought to be prevalent in tropical regions, such as Southeast Asia, Oceania and parts of the Indian subcontinent. These infections demonstrate the shortest relapse interval of approximately three weeks. In contrast North Indian, Madagascar and St. Elizabeth (United States and Southern Europe) strains follow a pattern of early relapses followed by a long interval before subsequent relapses, whereas the first relapse of the *hibernans* strains (Korea, Netherlands, Scandinavia, Central Russia) is delayed by 8–12 months [4]. Such variation in relapse patterns, the absolute risk of recurrence and the delay in clinical manifestations represent major confounding factors undermining the interpretation of primaquine efficacy.

The anti-malarial properties of primaquine, an 8-aminoquinoline, were first documented in 1946. The drug showed activity against both asexual and sexual blood stages of the parasite as well against the liver stage schizonts and hypnozoites [5-7]. Since the 1950s, clinicians and policy makers have relied on primaquine as the only licensed agent for the radical cure of *P. vivax*. However, despite over 60 years of continuous use there is still neither evidence nor consensus on the optimal dose or dosing regimen. Although primaquine pharmacokinetics have been well characterized in studies of healthy subjects and adult males with malaria, there are virtually no data available in young children or pregnant women, the most vulnerable populations in urgent need of an effective radical cure.

Primaquine can result in significant haemolysis particularly in those with glucose-6-phosphate dehydrogenase deficiency (G6PDd) [8,9]. G6PD deficiency is the most common heritable enzymopathy in the world, with a prevalence ranging from 2% to 40% [10]. Generally, the more severe the enzyme deficiency, the greater the severity of haemolysis; individuals with less than 10% of normal enzyme activity being at risk of life-threatening haemolysis even after a single dose of primaquine [11]. Individuals with milder variants may have negligible effects on exposure to primaquine [12]. In view of the risk of adverse reactions, primaquine dosing strategies are influenced more by concerns over toxicity than by their absolute efficacy. These concerns are particularly important in poorly resourced settings where routine G6PDd testing is not available. Early clinical studies demonstrated that the predominant determinant of therapeutic efficacy was the total dose of primaquine administered rather than the daily dosage or duration of therapy [13]. Some policy makers therefore attempt to mitigate the risk of serious harm by opting for a lower daily dose spread over 14-days or a single weekly dose for 8 weeks for G6PDd patients [14]. Ultimately the effectiveness of such regimens is dependent upon adherence, which is a function of a range of factors, particularly tolerability, the duration of therapy and supervision of drug administration.

The World Health Organization (WHO) guidelines for the radical cure of vivax malaria currently recommends the use of a daily dose of 0.25 mg/kg/day (3.5 mg/kg total dose) primaquine taken with food once daily, co-administered with chloroquine or artemisinin combination therapy (ACT) depending on chloroquine sensitivity in the region [15,16]. In South East Asia and Oceania, the same guidelines recommend an increase in the daily dose to 0.5 mg/kg (7.0 mg/kg total dose) in view of the high proportion of relapses. However there is a lack of data regarding the benefits of the higher dose, particularly from studies with prolonged follow up. In this paper we present a systematic review of the literature to highlight the diversity of methodologies that have been applied to anti-relapse clinical trials and to summarize the evidence of primaquine efficacy for different regimens across a range of epidemiological and geographical locations.

## Methods

### Literature search

A MEDLINE search was conducted for all studies on malaria using the search terms “malaria or *Plasmodium*” and all possible anti-malarial drugs; Additional file 1. This database was then filtered for clinical studies and studies containing “vivax” and “primaquine” in the title or abstract. In addition, the Cochrane Central Register of Controlled Trials (CENTRAL) was searched using the terms “(malaria or vivax) and primaquine”. Reference lists of previously published reviews and papers on primaquine were also screened for relevant studies in English.

### Data extraction

Data regarding study characteristics, recurrence and side effects were systematically extracted from the articles and entered into an EpiInfo^TM^ 3.5.1 database and analyzed using SPSS® Statistics vs19. Details of the study methodology including study site, inclusion and exclusion criteria, identification of clinical setting, randomization and blinding were extracted from all studies (Additional file 2). The details of the dosing regimen such as co-administered drug, primaquine dosing, frequency, duration and supervision of treatment were extracted for each arm of included studies. The dose of primaquine was calculated according to the total dose per kg assuming a 60 kg adult, and categorized as very low dose (≤2.5 mg/kg), low dose (>2.5 mg/kg- < 5.0 mg/kg) and high dose (≥ 5.0 mg/kg). Study sites were identified using Google Earth™ and GPS co-ordinates presented using GPS online visualizer [17].

### Statistical analysis

The risk of recurrence was calculated according to a per protocol analysis using the number of patients with recurrent *P. vivax* during follow up, divided by the number of patients followed until the study endpoint. The analysis of the risk of recurrence was recorded only once for each treatment arm, at the maximum period of follow up. Recurrence rates were then presented according to the total dose of primaquine stratified by geographical regions. Studies carried out in the USA recruited a mix of soldiers returning from the war in Korea, Vietnam and Somalia as well as induced malaria using Chesson and West Pakistan strains; for the purposes of analysis these were categorized according to origin of the infecting strain. Recurrence rates following anti-relapse treatment were categorized into three groups: “Adequate Responders” if there were less than 10% recurrence in studies with greater than 90 days follow up and “Poor responders” for studies with recurrence rate greater than or equal to 10% at any time during follow up. Studies with recurrence rates less than 10% but of short duration (< 90 days) were categorized as indeterminate. Studies with a control arm without primaquine were compared by Mantel-Haenszel odds ratios (OR), calculated using Review Manager (RevMan version 5.1. Copenhagen). Recurrence data after primaquine regimens at 1 month, 2–3 months, 4–6 months and 6 months were calculated using the Mann–Whitney *U* test, using SPSS vs19.0 for windows software (SPSS Inc., Chicago, Illinois).

### Nomenclature

Relapse refers to the phenomenon of activation of dormant *P. vivax* hypnozoites in the liver leading to a malarial attack. Recurrence, on the other hand, is a blanket term encompassing a repeat malarial infection which can be caused by either recrudescence, relapse or re-infection [18,19] There is currently no reliable way of distinguishing between the three alternatives causes of recurrence [20] . In this article results are reported with regard to recurrences of *P. vivax* malaria.

## Results

### Details of included studies

**Figure 1:**
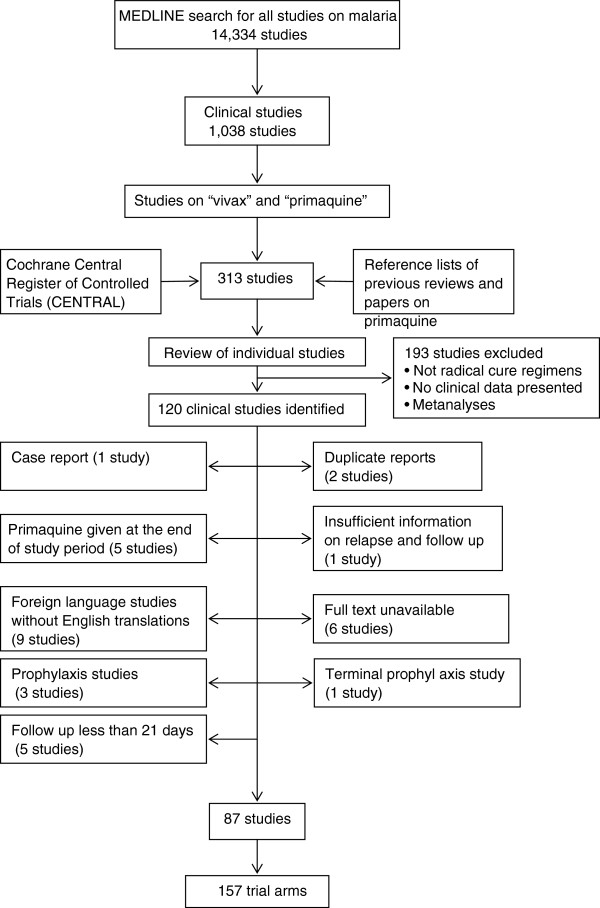
Profile of clinical studies included in the analysis.

**Figure 2:**
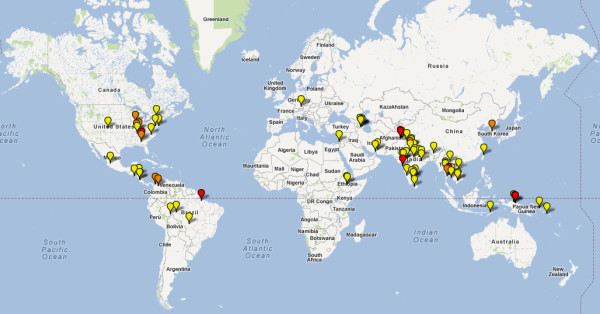
**Map of study sites of primaquine antirelapse studies.** Foot Note: One study (yellow icon); Two studies (orange icon), Three or more studies (red icon).

A total of 120 clinical trials of primaquine were identified, of which 33 studies were excluded for the reasons listed in Figure 1. Of the 87 clinical trials included in the analysis, 43 (49%) were conducted on or after 2000 (Additional file 3). The greatest number of trials came from Thailand (24 studies) followed by India (17 studies); see Figure 2. Three studies in Germany, Israel and Taiwan enrolled travelers to endemic countries returning with imported *P. vivax* malaria. Studies conducted in the United States recruited soldiers returning from wars in Korea (n = 3), Vietnam (n = 2) and Somalia (n = 2) or artificial inoculation of *P. vivax* in inmates of state penitentiaries (n = 7). None of the studies enrolled pregnant women. Details of the studies are provided in Additional files 4 and 5.

### Design of the reviewed trials

There was marked heterogeneity in the design of the clinical studies (Table 1) and dosing strategies of primaquine investigated (Additional file 4). In total 54 studies reported comparative drug trials, of which 30 (56%) were randomized. The median number of treatment arms in these comparative studies was 3 (range 2–9), with 18 studies having a control arm in which patients did not receive anti-relapse therapy. The remaining studies were either single arm cohort studies (n = 28) or retrospective (n = 5).

**Table 1:** Characteristics of selected studies

**Year of publication**	**No. of studies**
**≤1960**	8
**1961-70**	3
**1971-80**	10
**1981-90**	4
**1991-00**	24
**2001-10**	36
**2011**	2
**Study setting and participants**	**No. of studies**
**Community setting**	19
**In-hospital setting**	27
**Out-patient setting**	24
**Soldiers**	7
**Travellers**	3
**Volunteers (induced)**	7
**Follow up period**	**No. of studies**
**< 30 days**	27
**31-120 days**	11
**121-179 days**	3
**180 days**	13
**181-364 days**	14
**365 days**	8
**>365 days**	11

Age-related inclusion criteria were recorded for 59 studies, of which 33 (56%) actively excluded children less than 14 years old. Parasitaemia criteria were applied for enrolment in 13 (15%) studies, the threshold for inclusion varying from greater than 40 asexual parasites/μl to less than 100,000 parasites/μl. Only 23 (26%) studies reported actively testing and excluding patients with G6PD deficiency. In 31 studies, patients with a history of recent treatment were excluded, the interval since prior treatment varying from 48 hours to 2 months.

The median duration of follow up was 180 days, ranging from 28 to 2,555 days (Additional file 4), and differed markedly with the location of the studies. The majority of studies conducted in Southeast Asia and Oceania (Thailand, Indonesia, Vietnam and Papua New Guinea) were of short duration, 70% (21/30) with no more than a month of follow up. Conversely follow up was continued for 6 months or more in 80% (9/12) of the South American studies and 90% (19/21) of studies on the Indian subcontinent.

### Primaquine dosing regimens

Very low total dose primaquine was used in 44 (28%) of 156 treatment regimens, low dose in 82 regimens (53%) and high dose in 28 regimens (18%). In one study (two treatment arms) the dose of primaquine was not stated; see Additional file 5. Primaquine was administered within 15 days in 140 (90%) of treatment regimens. The remaining 16 treatment arms were administered weekly or on alternate days over 21 to 98 days in studies conducted in Indonesia (n = 4), Pakistan (n = 1), soldiers returning from Vietnam or Somalia (n = 3) or induced malaria with Chesson strain (n = 8). The supervision of administration was recorded in 51% (44/87) studies, with 81% (60/74) of treatment arms fully supervised, 15% (11/74) part supervised and 4% (3/74) unsupervised. In 114 (73%) treatment arms chloroquine was given as the accompanying schizontocidal therapy. Other schizontocides used included artesunate (n = 17), artemether-lumefantrine (n = 1), amodiaquine (n = 2), atovaquone/proguanil (n = 2), azithromycin (n = 1), sulfadoxine-pyrimethamine (n = 1), halofantrine (n = 1), mefloquine (n = 1), quinine (n = 4) and rifampicin (n = 1) and DB289 (n = 1). In one study the schizontocidal drug was not stated, and in another (with 2 treatment arms) patients received either chloroquine or quinine regimens. Primaquine was used as monotherapy in seven studies.

### Risk of recurrence

**Figure 3:**
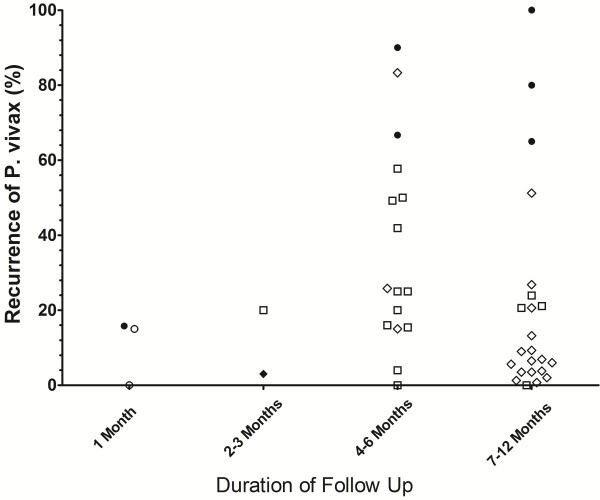
Risk of recurrence at the end of the study following very low dose primaquine.

**Figure 4:**
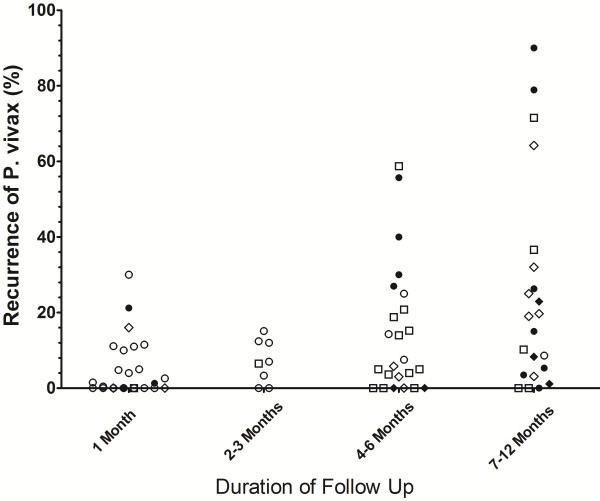
Risk of recurrence at the end of the study following low dose primaquine.

**Figure 5:**
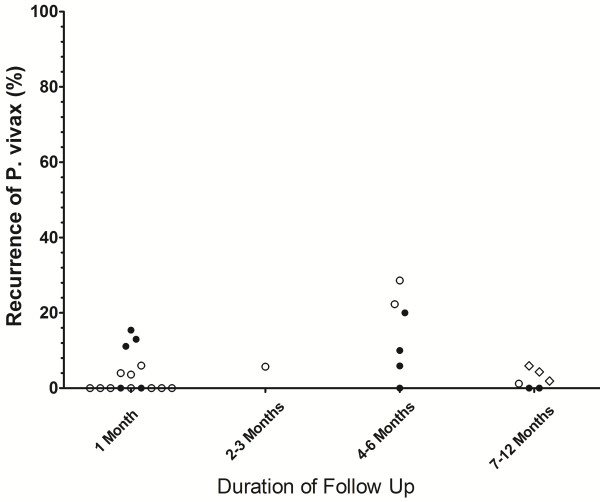
Risk of recurrence at the end of the study following high dose primaquine.

**Figure 6:**
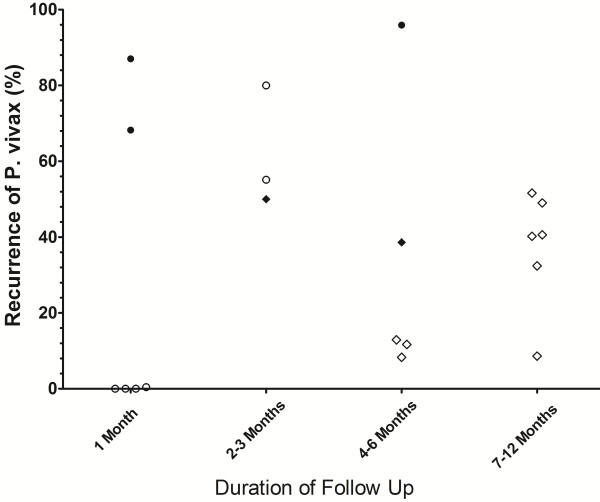
**Risk of recurrence at the end of the study in control arms in which no primaquine was given.** Footnotes for Figures 3, 4, 5 and 6: Indonesia and PNG [Closed Circles]; Thailand and Vietnam (Open Circles), South and Central America (Open Squares); Indian Subcontinent, Middle East and Horn of Africa (Open Diamonds); Korea and China (Closed Diamonds). USA studies of induced malaria and returning soldiers categorized according to origin of infecting strain. Full details are given in Additional file 5.

The risk of recurrence according to geographical location for different periods of follow up are presented in Figures 3, 4, 5, 6, with further details in Additional file 5. Studies using the very low dose regimen (Figure 3) were conducted mostly in the Indian subcontinent (19/44) and South America (16/44), with longer follow up (median 220 days; Range 28–1440). Across all regions, the median recurrence rate following very low dose primaquine was 15% (range 0-16%) at 1 month, 11.5% (range 3-20%) at 2–3 months, 25% (range 0–90) at 4–6 months, but fell to 9.2% (range 0-100%) at 7–12 months, reflecting almost exclusive representation of studies originating on the Indian subcontinent. In total 36% (16/44) of treatment arms were categorized as having an adequate response and 61% (27/44) having a poor response, with 1 study being indeterminate (Additional file 6).

Low dose primaquine was assessed in 82 treatment arms with a median recurrence of 0.9% (range 0-30%) at 1 month, 6.8% (range 0-15%) at 2–3 months, 6.7% (range 0-59%) at 4–6 months and 17% (range 0-90%) at 7–12 months (Figure 4). In total 32% (26/82) of treatment arms were categorized as having an adequate response, 44% (36/82) having a poor response, 24% (20/82) studies being indeterminate (Additional file 7). In Thai studies low dose primaquine following chloroquine was associated with a median recurrence rate of 0% [Range: 0–1.5] at one month, significantly lower than in patients who initially received artesunate; (median = 4.8% [Range: 4–11]; p = 0.009).

High dose primaquine was administered in 28 treatment arms from 17 studies, and was associated with a median risk of recurrence of 0% (Range: 0-15%) at 1 month (Figure 5). In the 16 treatment arms in which primaquine treatment was administered within 15 days the risk of recurrence at 1 month was 0% (Range: 0-6%) compared to 12% (Range: 0-15%) in the 11 studies in which primaquine was administered on alternate days or weekly. This difference was also apparent in studies with longer follow up: 0.6% (0–1.9%) at 7–12 months following short course regimens compared to 15% (0-29%) at 4–6 months following intermittently dosed regimens (Additional file 5). In total 29% (8/28) of treatment arms were categorized as having an adequate response, 25% (7/28) having a poor response, with 46% (13/28) of studies being indeterminate (Additional file 8). All of the seven primaquine treatments categorized as having a poor response were administered intermittently over a period of 27 days or more.

### Odds of *P. vivax* recurrence after treatment

**Figure 7:**
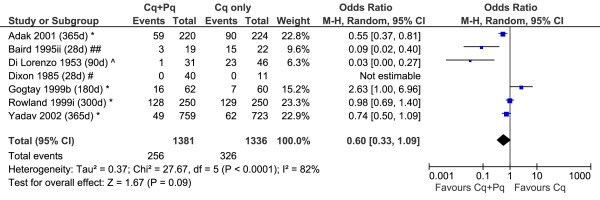
Forest plot of the effectiveness of very low dose primaquine in studies with a control arm.

**Figure 8:**
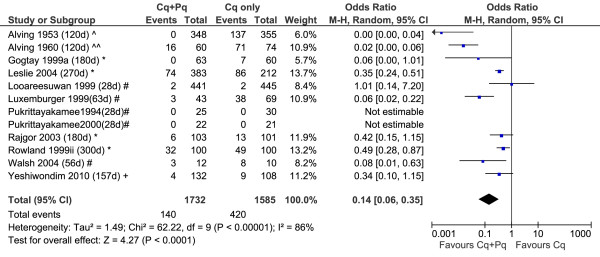
Forest plot of the effectiveness of low dose primaquine in studies with a control arm.

**Figure 9:**
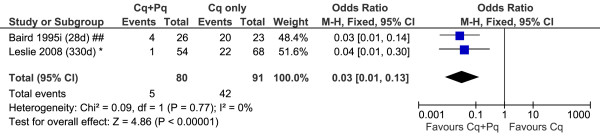
**Forest plot of the effectiveness of high dose primaquine in studies with a control arm.** Footnote for Figures 7, 8 and 9: * Indian subcontinent; ^ USA (Korea), ^^ USA (Chesson), # Thailand, ## Indonesia, + Ethiopia.

A total of 18 studies included patients recruited into control arms in which no primaquine was given; two of these studies had two control arms each; see Additional file 5 and Figures 7, 8, 9. The diversity of the risk of recurrence according to the duration of follow up and location of the study site is apparent from review of the control arms alone (see Figure 6). All but one of the six comparative studies of very low dose primaquine were conducted in the Indian subcontinent; the overall risk of recurrence did not differ significantly from patients who received no primaquine (OR = 0.60, [95%CI 0.33-1.09], p = 0.09), although there was marked heterogeneity between studies (I^2^ = 82%). Low dose primaquine regimens were significantly better than control regimens in 50% (6/12) of studies assessed (OR = 0.14 [95% CI 0.06-0.35], p < 0.001). Two studies demonstrate the high efficacy of high dose primaquine when compared with a control arm (OR = 0.03 [95%CI: 0.01-0.13]; p < 0.0001).

**Figure 10:**
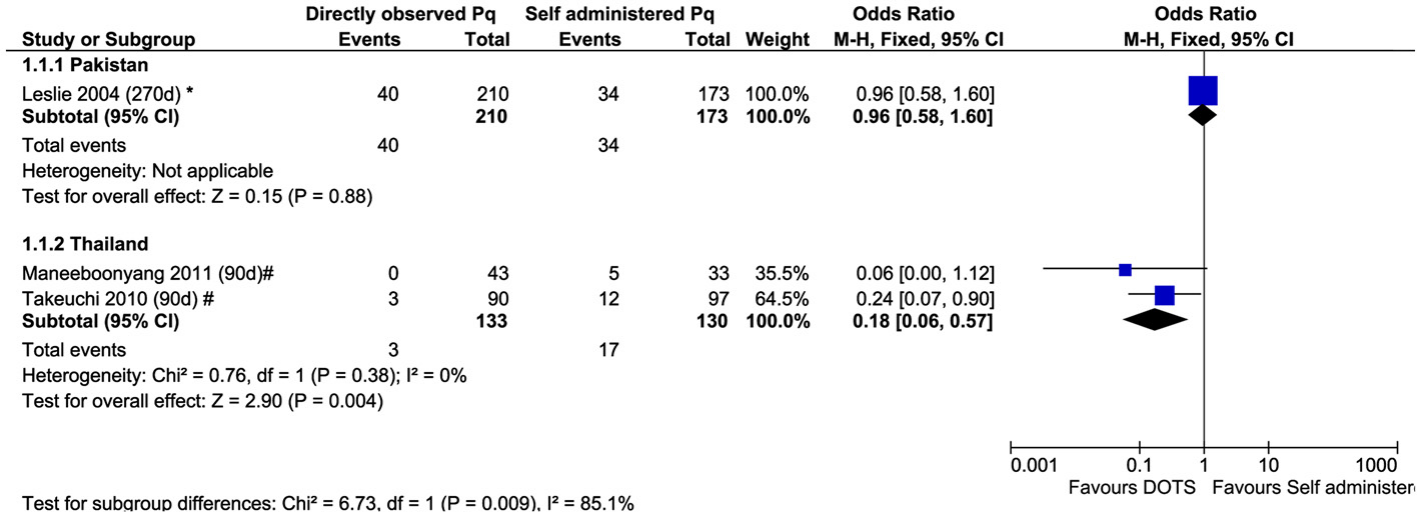
Forest plot of the effectiveness of directly observed treatment in primaquine therapy.

Three studies compared the risk of recurrence in patients administered primaquine under direct supervision with those receiving unsupervised treatment. In the two Thai studies recurrence rates of *P. vivax* were significantly lower in the supervised treatment arms (combined OR: 0.18 [95%CI: 0.06-0.57]) whereas in a study conducted in Pakistan there was no significant difference between treatment arms; see Figure 10.

### Safety data

Overall, 39 (48%) studies reported monitoring for adverse effects of treatment recruiting a total of 11,466 patients (6,986 with very low dose, 3,322 with low dose, 961 treated with high dose and 197 with the dose not stated). A total of 10 serious adverse events (SAE) were reported potentially related to primaquine toxicity; these are listed in Additional file 9. The overall rate of SAE was therefore 8.7 (95% CI: 4.2-16) per 10,000. Assuming denominators were all G6PD normal, the estimated rate of SAEs in G6PD normal individuals was 3.0 (95% CI: 0.1-16.8) per 10,000 following low dose primaquine and 10.4 (95% CI: 0.3-58) per 10,000 following high dose primaquine. No deaths were reported in any study.

## Discussion

Despite the recognition that *P. vivax* constitutes a major threat to human health and the goal of malaria elimination, knowledge of the optimal treatment strategies for this parasite has changed little over 60 years. Newer drugs with activity against liver stages of the parasite are under development [21], but these are, at best, 5 to 10 years from reaching the global market. In the meantime better strategies need to be devised for delivering safe and effective antirelapse therapy using primaquine.

The 2010 WHO guidelines recommend a total primaquine dose of 3.5 mg/kg dose for the radical cure of *P. vivax*, whilst acknowledging that in Southeast Asia and Oceania a higher dose (7.0 mg/kg total dose) may be warranted [15]. However, the evidence from which these recommendations are derived is limited. Malaria control programs prioritize acute anti-malarial policy towards eliminating the initial threat to the patient (i.e. from blood stage parasites), rather than preventing future recurrences from hypnozoites. The most important factor for primaquine treatment policy is safety rather than efficacy [16]. However the benefits of radical cure may not be appreciated. Early studies in soldiers returning from the Korean War demonstrated that African-Americans were particularly prone to primaquine-induced haemolysis [22,23]. This risk could be mitigated by reducing the dose and, in the Korean strains of *P. vivax* tested, this appeared to be feasible without compromising efficacy.

The main findings of this review of the literature are presented in Additional file 10. The plots presented in Figures 3 and 4 highlight the wide range of primaquine efficacy presented by published clinical trials. Both very low dose and low dose primaquine were vulnerable to high rates of recurrence of *P. vivax* (Figure 3 and 4), whereas the use of high dose primaquine was associated more often with acceptable efficacy [16]. The treatment efficacy of high dose regimens appeared significantly better when regimens are given over 14 days rather than spread out over alternate days or weekly dosing. However such comparisons need to be interpreted with caution, since they are confounded by marked heterogeneity in study design, dose regimens, and idiosyncrasies of the endemic setting. The frequency and the timing of relapses are determined by sporozoite inoculum and parasite relapse phenotype and this results in huge regional variation in the absolute risk of recurrence independent of primaquine efficacy [4,24]. Goller et al. applied a multivariate model to compare relapses in studies conducted in Brazil, India and Thailand demonstrating that after controlling for year of study, study design and dose of primaquine, the overall risk of relapse in Thailand was 10 times that in India and twice that in Brazil [25]. Alternatively primaquine efficacy can be gauged by comparison with a control arm in which patients receive no primaquine, thus controlling for background reinfection and relapse patterns. In their 2007 Cochrane review, Galappaththy and colleagues used such an approach to control for differences in the background rates of infection, concluding that the 5-day primaquine regimen was ineffective and that a 14-day low dose regimen was 13-fold better compared to the five-day regimen [26].

Age is a major determinant of recurrent infection the risk of recurrence falling significantly as age increases. Although this may reflect exposure to new infections age is also a surrogate marker of immunity. Young patients are at greatest risk of symptomatic recurrent infections and morbidity associated with repeated vivax parasitaemia [27,28], and hence should be a priority for optimizing therapeutic interventions; in practice however they are often excluded from clinical trials. Pharmacokinetics can differ markedly in children and simply extrapolating dosing strategies from adults can result in systematic under dosing as was shown convincingly with the use of antifolates in *P. falciparum*[29]. Furthermore studies in adult populations can overestimate true primaquine efficacy since these patients are more likely to control *P. vivax* parasitaemia, remain asymptomatic or even clear the infection without treatment.

Based on the current analysis, the key elements required for the design of informative clinical studies of relapse prevention are presented in Additional file 11. The duration of follow up is a crucial component of the study design. In Southeast Asia and Oceania, early relapse occurs in a high proportion of patients and marked differences in antirelapse efficacy can be observed with short duration of follow up. Only five studies from Southeast Asia and Oceania (Thailand, Indonesia, Vietnam and Papua New Guinea) continued follow-up beyond three months. In areas with a more temperate climate the risk of relapse is lower and relapse tends to occur late, often months after the initial infection. In such circumstances, short-term follow-up will fail to pick up treatment failures, falsely elevating efficacy estimates. Such a situation occurred in India, where a 5-dose primaquine regimen was long heralded as an appropriate treatment strategy, but actually reflected an intrinsic low absolute risk of relapse; the majority of relapses in adults being subclinical [29,30].

Early recurrence of *P. vivax* (within 21 to 63 days) is a function of the efficacy of the initial schizontocidal treatment in clearing the initial blood stage biomass as well as the post treatment prophylaxis afforded by slowly eliminated drugs [31]. This is highlighted in studies from Thailand in which almost none of the patients receiving chloroquine plus low dose primaquine had a recurrence within one month, a reflection of the high efficacy chloroquine in this location and its prolonged suppressive activity on relapsing parasites [32,33]. In contrast in the same location patients receiving primaquine combined with short acting but effective artesunate regimens, had a risk of recurrence of between 4 and 15%, likely a better indication of the underlying primaquine efficacy [34]. Short duration studies of primaquine with slowly eliminated efficacious schizontocidial treatments are therefore of limited benefit in defining anti-relapse efficacy. Studies with long duration of follow up capture a greater proportion of relapses, but are vulnerable to confounding from heterologous new infections. The rise of chloroquine resistance adds an additional complication of partial clearance of the initial blood stage biomass, in which early recrudescence can be misclassified as a relapsing infection. Currently, no reliable method exists to differentiate relapses from re-infections and recrudescent infections.

Current guidelines recommend a 14 day course of primaquine administered either once or twice daily to reduce the risk of haemolysis and improve tolerability from gastrointestinal disturbance. Advocating such prolonged courses of treatment can result in significant problems with adherence, as highlighted by the markedly greater risk of recurrence in Thai patients receiving unsupervised treatment compared to those in whom a complete treatment course was confirmed [35,36]. Poor adherence to a 14-day course of unsupervised primaquine is likely to have a major impact on its public health benefit. Short-course, high dose regimens have potential to increase patient adherence and thus effectiveness [34], but appropriately powered prospective multi-centered randomized controlled trials are needed to assess the efficacy and effectiveness of such alternative radical curative regimens against current best practice. The current methodology for primaquine treatment trials can be improved and should be harmonized to facilitate comparison between different sites. The factors highlighted in this review suggest that a minimum of six months and perhaps even 12 months follow up is required. Relapse is often a recurrent process rather than a single event, hence the analysis of such trials should focus on the incidence of treatment failure rather than the cumulative risk of the first event, which is the standard outcome measure for drug efficacy studies in *P. falciparum* malaria. Such cohort studies have a similar rationale as vaccine intervention studies in which efficacy is interpreted against a control intervention to account for new infections and variation in relapse patterns. The use of a control arm introduces a number of important ethical considerations. At some sites schizontocidal treatment alone is standard of care – governments fearing more harm than benefit by giving primaquine to patients without prior G6PDd. Although adverse events were not reported consistently in the clinical studies reviewed, what data that was available suggested the rate of SAEs was 3 per 10,000 exposures to low dose primaquine and 10.4 per 10,000 exposures to high dose primaquine. These figures are comparable to a risk of 4.8 to 8.2 per 10,000 (1 per 2089 and 1 per 1217) severe neuropsychiatric reactions following 15 and 25 mg/kg dose of mefloquine respectively [37].

Most endemic countries recommend, at least in principle, use of primaquine with expressed warnings on its potential toxicity in G6PDd patients. Several studies highlight that even in areas where primaquine is recommended, in practice it is prescribed rarely and even when given, adherence and thus effectiveness is often poor [38,39]. Investigation of different primaquine regimens must also include appropriate measures of tolerability and be able to quantify the risk of severe adverse events, especially in patients with different variants of G6PDd. Here again a control arm is vital, since only then can the consequences of recurrent episodes of malaria on the health and well being of the patient be assessed (Additional file 11).

Clinical research priorities for optimizing the radical cure of *P. vivax* are presented in Additional file 12. In populations with high rates of G6PDd, particularly those with variants at greatest risk of haemolysis, a critical hurdle for the safe deployment of primaquine will be the development of a rapid affordable point of care test. Cases of severe haemolysis following a single dose of primaquine can have a negative impact not only for the patient but also for public confidence in public health interventions [40,41]. The development of newer and inexpensive methods for field-testing of G6PD deficiency should go hand in hand with studies of primaquine effectiveness. Reliable exclusion of patients at risk of severe harm opens the possibility for safer and more effective deployment of high dose regimens. If the elimination of vivax is to be achieved, a co-ordinate effort is needed to provide the evidence across the spectrum of the vivax endemic world, from which to rationalize and deploy antirelapse therapy in a safe and effective manner.

## Competing interest

JKB serves in an unpaid capacity on a board advising GSK on the clinical development of tafenoquine, a therapy aimed at replacing primaquine. He accepts travel support from GSK in connection with that appointment. All other authors: no conflicts. NJW is co-chairman of the WHO anti-malarial treatment guidelines committee. All other authors declare that they have no conflicts of interest.

## Authors’ contribution

GKJ and RNP designed the study and searched the relevant literature. GKJ extracted and analyzed the clinical data and wrote the first draft of the manuscript. All authors appraised and revised the manuscript. All authors gave final approval for submission of the manuscript.

## Supplementary Material

Additional file 1 Search terms used for the literature review. Click here for file

Additional file 2 Extracted data. Click here for file

Additional file 3 Complete list of all articles included in the analysis. Click here for file

Additional file 4 Study design of articles included in the analysis. Click here for file

Additional file 5 Recurrence rates reported for all primaquine treatment arms. Click here for file

Additional file 6 **Map of studies documenting the effectiveness of very low dose primaquine.** Footnote: Adequate Responders (yellow icon): Recurrence rate < 10% in studies with greater than 6 weeks follow up; Poor responders (red icons): recurrence rate >10% at any time during follow up. Studies of returning US soldiers are placed at country of origin. Indeterminate studies and studies of induced malaria have been excluded. Click here for file

Additional file 7 **Map of studies documenting the effectiveness of low dose primaquine.** Footnote: Adequate Responders (yellow icon): Recurrence rate < 10% in studies with greater than 6 weeks follow up; Poor responders (red icons): recurrence rate >10% at any time during follow up. Studies of returning US soldiers are placed at country of origin. Indeterminate studies and studies of induced malaria have been excluded. Click here for file

Additional file 8 **Map of studies documenting the effectiveness of high dose primaquine. **Footnote: Adequate Responders (yellow icon): Recurrence rate < 10% in studies with greater than 6 weeks follow up; Poor responders (red icons): recurrence rate >10% at any time during follow up. Studies of returning US soldiers are placed at country of origin. Indeterminate studies and studies of induced malaria have been excluded. Click here for file

Additional file 9 Severe adverse events reported. Click here for file

Additional file 10 Main findings of the literature review. Click here for file

Additional file 11 Key components in the design of studies of relapse prevention by primaquine in vivax malaria. Click here for file

Additional file 12 Clinical research priorities for optimising the radical cure of P. vivax. Click here for file
