# Supplementary figures and images for: Primaquine radical cure of Plasmodium vivax: a critical review of the literature
Source: Malar J. 2012 Aug 17;11:280. doi: 10.1186/1475-2875-11-280 (PMC3489597; doi:10.1186/1475-2875-11-280)

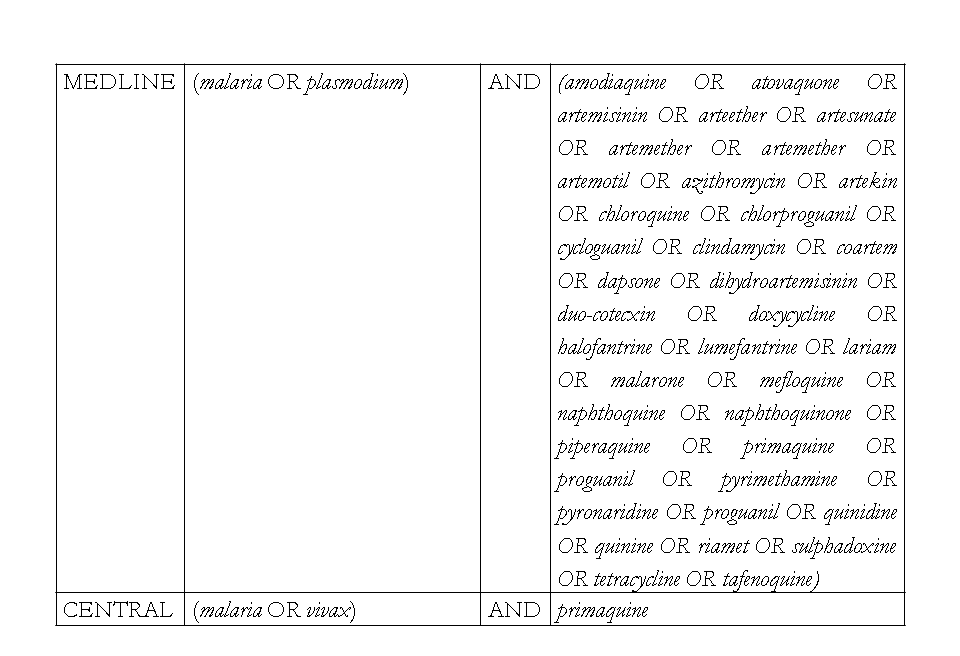

Supplement: Additional file 1 — Search terms used for the literature review. [file 1475-2875-11-280-S1.tiff]

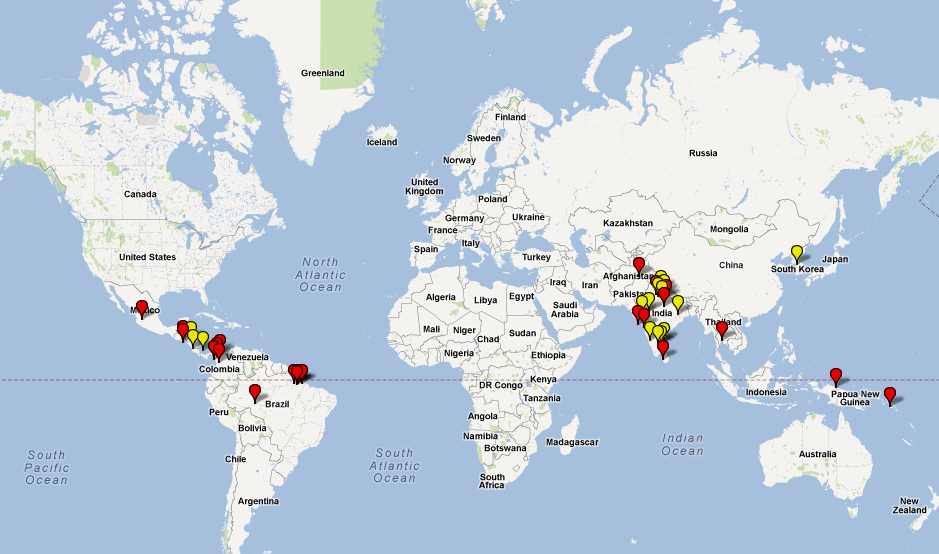

Supplement: Additional file 6 — Map of studies documenting the effectiveness of very low dose primaquine. Footnote: Adequate Responders (yellow icon): Recurrence rate < 10% in studies with greater than 6 weeks follow up; Poor responders (red icons): recurrence rate >10% at any time during follow up. Studies of returning US soldiers are placed at country of origin. Indeterminate studies and studies of induced malaria have been excluded. [file 1475-2875-11-280-S6.tiff]

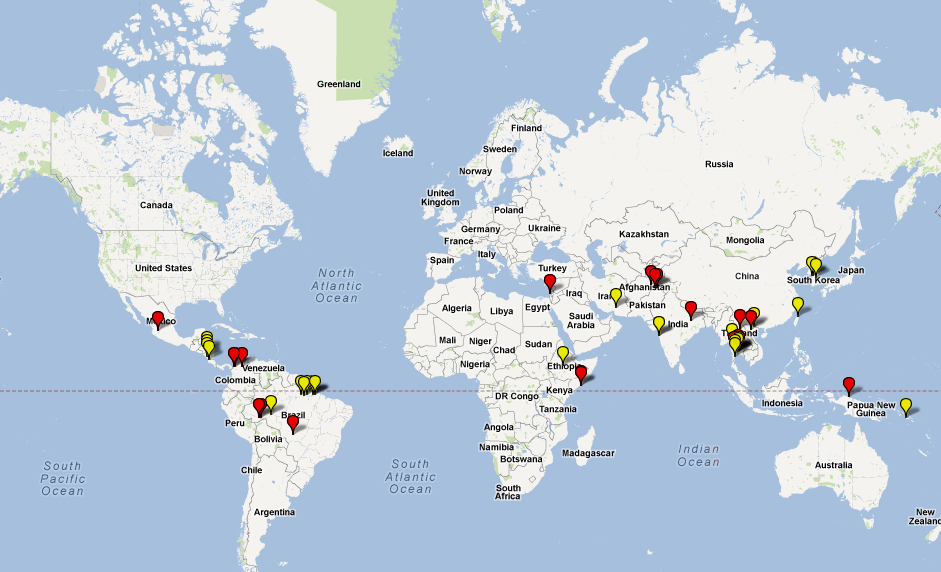

Supplement: Additional file 7 — Map of studies documenting the effectiveness of low dose primaquine. Footnote: Adequate Responders (yellow icon): Recurrence rate < 10% in studies with greater than 6 weeks follow up; Poor responders (red icons): recurrence rate >10% at any time during follow up. Studies of returning US soldiers are placed at country of origin. Indeterminate studies and studies of induced malaria have been excluded. [file 1475-2875-11-280-S7.tiff]

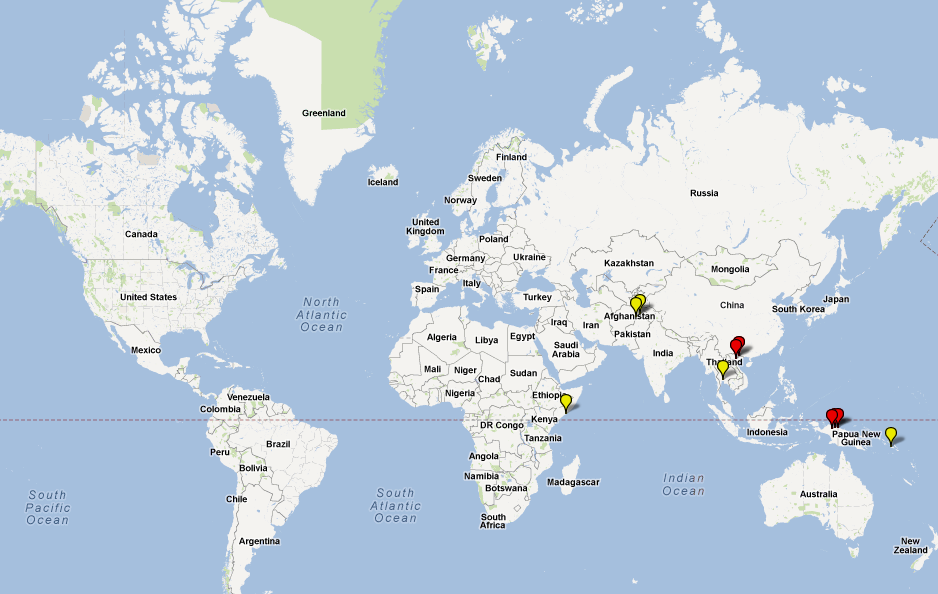

Supplement: Additional file 8 — Map of studies documenting the effectiveness of high dose primaquine. Footnote: Adequate Responders (yellow icon): Recurrence rate < 10% in studies with greater than 6 weeks follow up; Poor responders (red icons): recurrence rate >10% at any time during follow up. Studies of returning US soldiers are placed at country of origin. Indeterminate studies and studies of induced malaria have been excluded. [file 1475-2875-11-280-S8.tiff]
