# Supplementary material for: Primaquine radical cure of Plasmodium vivax: a critical review of the literature
Source: Malar J. 2012 Aug 17;11:280. doi: 10.1186/1475-2875-11-280 (PMC3489597; doi:10.1186/1475-2875-11-280)
Supplement: Additional file 3 — Complete list of all articles included in the analysis. [file 1475-2875-11-280-S3.pdf]

Additional File 3: Study References

Authors	Year	Title	Journal	Volume	Issue	Pages	Reason for Exclusion
Edgcomb et. al.	1950	Primaquine, SN 13272, a new curative agent in vivax malaria; a preliminary report	Journal National Malaria Society	9	4	285-92	Full text not available
Alving et. al.	1953	Korean vivax malaria. II. Curative treatment with pamaquine and primaquine	The American journal of tropical medicine and hygiene	2	6	970-6	
Coatney et. al.	1953	Korean vivax malaria. V. Cure of the infection by primaquine administered during long-term latency	The American journal of tropical medicine and hygiene	2	6	985-8	
Cooper et. al.	1953	Studies in human malaria. XXXI. Comparison of primaquine, isopentaquine, SN-3883, and pamaquine as curative agents against Chesson strain vivax malaria	The American journal of tropical medicine and hygiene	2	6	949-57	
Di Lorenzo et. al.	1953	Korean vivax malaria. IV. Curative effect of 15 milligrams of primaquine daily for 7 days	The American journal of tropical medicine and hygiene	2	6	983-4	
Singh et. al.	1953	Preliminary studies on 8-aminoquinolines	Indian journal of malariology	7		289-94	Full text not available
Thaeler et. al.	1953	A clinical study of primaquine (S. N. 13,272) in the treatment of malaria among the Miskito Indians of Nicaragua	The American journal of tropical medicine and hygiene	2	6	989-99	
Singh et. al.	1954	Antirelapse treatment with primaquine and pyrimethamine	Indian journal of malariology	8	2	127-36	Full text not available
Alving et. al.	1955	Potentiation of the curative action of primaquine in vivax malaria by quinine and chloroquine	The Journal of laboratory and clinical medicine	46	2	301-6	
Alving et. al.	1960	Mitigation of the haemolytic effect of primaquine and enhancement of its action against exoerythrocytic forms of the Chesson strain of Piasmodium vivax by	Bulletin of the World Health Organization	22		621-31	
Basavaraj et. al.	1960	Observations on the treatment of 678 malaria cases with primaquine in an area free from maiaria transmission in Mysore State, India	Indian journal of malariology	14		269-81	
Diwan et. al.	1962	Primaquin Trials as Antirelapse (Radical) Treatment for Malaria conducted in National Malaria Eradication Programme Units (East) Meerut and Mathura (Uttar Pradesh)	Bulletin of the National Society of India for Malaria and Other Mosquito Borne Diseases	10	2	91-102	Full text not available
Mendoza et. al.	1963	Comparative study of two regimens of radical treatment of vivax malaria in Mexico	WHO	527.65			
Martelo et. al.	1969	Malaria in American soldiers	Archives of internal medicine	123	4	383-7	
Fisher et. al.	1970	Malaria in soldiers returning from Vietnam. Epidemiologic, therapeutic, and clinical studies	The American journal of tropical medicine and hygiene	19	1	27-39	
Charoenlarp et. al.	1973	Relapses of vivax malaria after a conventional course of primaquine and chloroquine: report of 2 cases	The Southeast Asian journal of tropical medicine and public health	4	1	135-7	Full text not available
Contacos et. al.	1973	Five day primaquine therapy--an evaluation of radical curative activity against vivax malaria infection	The American journal of tropical medicine and hygiene	22	6	693-5	
Sharma et. al.	1973	Effectiveness of drug schedule being followed under the National Malaria Eradication Programme, India, for radical cure of vivax malaria cases.	Journal of Communicable Diseases	5		167-74	
Contacos et. al.	1974	Combined chloroquine-primaquine therapy against vivax malaria	The American journal of tropical medicine and hygiene	23	2	310-2	
Kaplan et. al.	1974	Improved therapy for Vietnam acquired vivax malaria	Military medicine	141	6	444-8	
Miller et. al.	1974	Sensitivity of four Central American strains of Plasmodium vivax to primaquine	The American journal of tropical medicine and hygiene	23	2	309-10	
Clyde et. al.	1977	Radical cure of Chesson strain vivax malaria in man by 7, not 14, days of treatment with primaquine	The American journal of tropical medicine and hygiene	26	3	562-3	
Roy et. al.	1977	Efficacy of 5-day radical treatment of P. vivax infection in Tamil Nadu	The Indian journal of medical research	65	5	652-6	
Saint-Yves et. al.	1977	Comparison of treatment schedules for Plasmodium vivax infections in the Solomon Islands	Papua and New Guinea medical journal	20	2	62-5	

Additional File 3: Study References

Authors	Year	Title	Journal	Volume	Issue	Pages	Reason for Exclusion
Cedillos et. al.	1978	Field evaluation of primaquine in the control of Plasmodium vivax	The American journal of tropical medicine and hygiene	27	3	466-72	
Roy et. al.	1979	Results of 5-day course of radical treatment of Plasmodium vivax in six districts of Tamil Nadu	The Indian journal of medical research	69		939-43	
Kondrashin et. al.	1981	Comparative studies on responses to 5-day treatment with primaquine of indigenous and imported cases of P. vivax in Nepal in 1974-76	Nepal Medical Association Journal	19		06-015	Full text not available
Appavoo et. al.	1984	Results of 3-day radical treatment of Plasmodium vivax in North Arcot and South Arcot Districts of Tamil Nadu	Indian journal of malariology	21	1	21-Apr	
Lapierre et. al.	1984	Drug resistance of Plasmodium falciparum and Plasmodium vivax strains in Cambodia (Cardamone Massif). Morphological characteristics of Plasmodium vivax	Medecine tropicale : revue du Corps de sante colonial	44	4	339-49	No translation available
Dixon et. al.	1985	A clinical trial of mefloquine in the treatment of Plasmodium vivax malaria	The American journal of tropical medicine and hygiene	34	3	435-7	
Harinasuta et. al.	1985	Trials of mefloquine in vivax and of mefloquine plus 'fansidar' in falciparum malaria	Lancet	1	8434	885-8	Insufficient information
Ohtomo et. al.	1987	Clinical evaluation of antimalarial regimens in Japan	Zentralblatt fur Bakteriologie Mikrobiologie und Hygiene. Series A Medical microbiology infectious diseases virology	264	3-Apr	513-20	No translation available
Rombo et. al.	1987	Seven patients with relapses of Plasmodium vivax or P. ovale despite primaquine treatment	Tropical medicine and parasitology : official organ of Deutsche Tropenmedizinische Gesellschaft and of Deutsche	38	1	49-50	No translation available
Arias et. al.	1989	Low response of Colombian strains of Plasmodium vivax to classical antimalarial therapy	Tropical medicine and parasitology : official organ of Deutsche Tropenmedizinische Gesellschaft and of Deutsche	40	1	21-Mar	No translation available
Sinha et. al.	1989	Efficacy of 5 day radical treatment of primaquine in Plasmodium vivax cases at the BHEL industrial complex, Hardwar (U.P.)	Indian journal of malariology	26	2	83-6	
Singh et. al.	1990	Radical treatment of vivax malaria in Madhya Pradesh, India	Indian journal of malariology	27	1	55-6	
Tanabe et. al.	1990	Clinical evaluation of antimalarial drugs	Kansenshogaku zasshi. The Journal of the Japanese Association for Infectious Diseases	64	6	668-73	No translation available
Boulos et. al.	1991	Frequency of malaria relapse due to Plasmodium vivax in a non-endemic region (Sao Paulo, Brazil)	Revista do Instituto de Medicina Tropical de Sao Paulo	33	2	143-6	No translation available
Prasad et. al.	1991	Relapse/reinfection patterns of Plasmodium vivax infection: a four year study	The Southeast Asian journal of tropical medicine and public health	22	4	499-503	
Ronn et. al.	1993	Recurrence problems with preventive primaquine treatment in patients with malaria	Ugeskrift for laeger	155	48	3901-4	No translation available
Bunnag et. al.	1994	High dose of primaquine in primaquine resistant vivax malaria	Transactions of the Royal Society of Tropical Medicine and Hygiene	88	2	218-9	
Pukrittayakamee et. al.	1994a	Antimalarial effects of rifampin in Plasmodium vivax malaria	Antimicrobial agents and chemotherapy	38	3	511-4	
Pukrittayakamee et. al.	1994b	Blood stage antimalarial efficacy of primaquine in Plasmodium vivax malaria	The Journal of infectious diseases	169	4	932-5	
Baird et. al.	1995	Treatment of chloroquine-resistant Plasmodium vivax with chloroquine and primaquine or halofantrine	The Journal of infectious diseases	171	6	1678-82	
Jelinek et. al.	1995	Long-term efficacy of primaquine in the treatment of vivax malaria in nonimmune travelers	The American journal of tropical medicine and hygiene	52	4	322-4	
Tan-ariya et. al.	1995	Clinical response and susceptibility in vitro of Plasmodium vivax to the standard regimen of chloroquine in Thailand	Transactions of the Royal Society of Tropical Medicine and Hygiene	89	4	426-9	
Phillips et. al.	1996	Failure of combined chloroquine and high-dose primaquine therapy for Plasmodium vivax malaria acquired in Guyana, South America	Clinical infectious diseases : an official publication of the Infectious Diseases Society of America	23	5	1171-3	Case report
Srivastava et. al.	1996	Studies on Plasmodium vivax relapse pattern in Kheda district, Gujarat	Indian journal of malariology	33	4	173-9	

Additional File 3: Study References

Authors	Year	Title	Journal	Volume	Issue	Pages	Reason for Exclusion
Fryauff et. al.	1997	Halofantrine and primaquine for radical cure of malaria in Irian Jaya, Indonesia	Annals of tropical medicine and parasitology	91	1	Jul-16	
Smoak et. al.	1997	Plasmodium vivax infections in U.S. Army troops: failure of primaquine to prevent relapse in studies from Somalia	The American journal of tropical medicine and hygiene	56	2	231-4	
Gogtay et. al.	1998	A 5 days primaquine regimen as anti-relapse therapy for Plasmodium vivax	Transactions of the Royal Society of Tropical Medicine and Hygiene	92	3	341	
Pinto et. al.	1998	Clinical efficacy of four schemes for vivax malaria treatment in children	Jornal de pediatria	74	3	222-7	No translation available
Soto et. al.	1998	Primaquine prophylaxis against malaria in nonimmune Colombian soldiers: efficacy and toxicity. A randomized, double-blind, placebo-controlled trial	Annals of internal medicine	129	3	241-4	
Fang et. al.	1999	Imported malaria: successful treatment of 31 patients in the era of chloroquine resistance	Journal of the Formosan Medical Association = Taiwan yi zhi	98	10	683-7	
Gogtay et. al.	1999	Efficacies of 5- and 14-day primaquine regimens in the prevention of relapses in Plasmodium vivax infections	Annals of tropical medicine and parasitology	93	8	809-12	
Li et. al.	1999	Observation on efficacy of artemether compound against vivax malaria	Zhongguo ji sheng chong xue yu ji sheng chong bing za zhi = Chinese journal of parasitology & parasitic diseases	17	3	175-7	
Looareesuwan et. al.	1999a	Chloroquine sensitivity of Plasmodium vivax in Thailand	Annals of tropical medicine and parasitology	93	3	225-30	
Looareesuwan et. al.	1999b	Atovaquone and proguanil hydrochloride followed by primaquine for treatment of Plasmodium vivax malaria in Thailand	Transactions of the Royal Society of Tropical Medicine and Hygiene	93	6	637-40	
Luxemburger et. al.	1999	Treatment of vivax malaria on the western border of Thailand	Transactions of the Royal Society of Tropical Medicine and Hygiene	93	4	433-8	
Rowland et. al.	1999	Randomized controlled trials of 5- and 14-days primaquine therapy against relapses of vivax malaria in an Afghan refugee settlement in Pakistan	Transactions of the Royal Society of Tropical Medicine and Hygiene	93	6	641-3	
Soto et. al.	1999	Double-blind, randomized, placebo-controlled assessment of chloroquine/primaquine prophylaxis for malaria in nonimmune Colombian soldiers	Clinical infectious diseases : an official publication of the Infectious Diseases Society of America	29	1	199-201	Prophylaxis study
Wilairatana et. al.	1999	Efficacy of primaquine regimens for primaquine-resistant Plasmodium vivax malaria in Thailand	The American journal of tropical medicine and hygiene	61	6	973-7	
Bergonzoli et. al.	2000	Therapeutic efficacy of different antimalarial regimens in the Costa Rica-Nicaragua border region	Revista panamericana de salud publica = Pan American journal of public health	7	6	366-70	
Kitchener et. al.	2000	Malaria in the Australian Defence Force during and after participation in the International Force in East Timor (INTERFET)	The Medical journal of Australia	173	11-Dec	583-5	Prophylaxis study
Pukrittayakamee et. al.	2000	Therapeutic responses to different antimalarial drugs in vivax malaria	Antimicrobial agents and chemotherapy	44	6	1680-5	
Schwartz et. al.	2000	Short report: a consideration of primaquine dose adjustment for radical cure of Plasmodium vivax malaria	The American journal of tropical medicine and hygiene	62	3	393-5	
Singh et. al.	2000	Emergence of chloroquine-resistant vivax malaria in south Bihar (India)	Transactions of the Royal Society of Tropical Medicine and Hygiene	94	3	327	
Villalobos-Salcedo et. al.	2000	In-vivo sensitivity of Plasmodium vivax isolates from Rond nia (western Amazon region, Brazil) to regimens including chloroquine and primaquine	Annals of tropical medicine and parasitology	94	8	749-58	
Abdon et. al.	2001	Assessment of the response to reduced treatment schemes for vivax malaria	Revista da Sociedade Brasileira de Medicina Tropical	34	4	343-8	
Adak et. al.	2001	Plasmodium vivax polymorphism in a clinical drug trial	Clinical and diagnostic laboratory immunology	8	5	891-4	
Buchachart et. al.	2001	Effect of primaquine standard dose (15 mg/day for 14 days) in the treatment of vivax malaria patients in Thailand	The Southeast Asian journal of tropical medicine and public health	32	4	720-6	
Dua et. al.	2001	Plasmodium vivax relapses after 5 days of primaquine treatment, in some industrial complexes of India	Annals of tropical medicine and parasitology	95	7	655-9	

Additional File 3: Study References

Authors	Year	Title	Journal	Volume	Issue	Pages	Reason for Exclusion
Duarte et. al.	2001	Association of subtherapeutic dosages of a standard drug regimen with failures in preventing relapses of vivax malaria	The American journal of tropical medicine and hygiene	65	5	471-6	
Soto et. al.	2001	Plasmodium vivax clinically resistant to chloroquine in Colombia	The American journal of tropical medicine and hygiene	65	2	90-3	Primaquine given at end of follow up
Taylor et. al.	2001	Chloroquine/doxycycline combination versus chloroquine alone, and doxycycline alone for the treatment of Plasmodium falciparum and Plasmodium vivax malaria in	The American journal of tropical medicine and hygiene	64	5-Jun	223-8	Primaquine given at end of follow up
Valecha et. al.	2001	Comparative antirelapse efficacy of CDRI compound 80/53 (Bulaquine) vs primaquine in double blind clinical trial	Current Science	80		561-563	Details same as Adak-2001
Lacy et. al.	2002	Atovaquone/proguanil therapy for Plasmodium falciparum and Plasmodium vivax malaria in Indonesians who lack clinical immunity	Clinical infectious diseases : an official publication of the Infectious Diseases Society of America	35	9	e92-5	
Congpuong et. al.	2002	Sensitivity of Plasmodium vivax to chloroquine in Sa Kaeo Province, Thailand	Acta tropica	83	2	117-21	
Hamedi et. al.	2002	Plasmodium vivax malaria in Southeast Iran in 1999-2001: establishing the response to chloroquine in vitro and in vivo	The Southeast Asian journal of tropical medicine and public health	33	3	512-8	
Yadav et. al.	2002	Radical curative efficacy of five-day regimen of primaquine for treatment of Plasmodium vivax malaria in India	The Journal of parasitology	88	5	1042-4	
Da Silva et. al.	2003	Short course schemes for vivax malaria treatment	Revista da Sociedade Brasileira de Medicina Tropical	36	2	235-9	
Fernandopulle et. al.	2003	Efficacy of a five-day course of primaquine in preventing relapses in Plasmodium vivax malaria--a pilot study	The Ceylon medical journal	48	1	32	
Machado et. al.	2003	Correlation between Plasmodium vivax variants in Belem, Para State, Brazil and symptoms and clearance of parasitaemia	The Brazilian journal of infectious diseases : an official publication of the Brazilian Society of Infectious Diseases	7	3	175-7	
Rajgor et. al.	2003	Efficacy of a 14-day primaquine regimen in preventing relapses in patients with Plasmodium vivax malaria in Mumbai, India	Transactions of the Royal Society of Tropical Medicine and Hygiene	97	4	438-40	
Silachamroon et. al.	2003	Clinical trial of oral artesunate with or without high-dose primaquine for the treatment of vivax malaria in Thailand	The American journal of tropical medicine and hygiene	69	1	14-Aug	
Valibayov et. al.	2003	Clinical efficacy of chloroquine followed by primaquine for Plasmodium vivax treatment in Azerbaijan	Acta tropica	88	1	99-102	
Walsh et. al.	2004	Randomized trial of 3-dose regimens of tafenoquine (WR238605) versus low-dose primaquine for preventing Plasmodium vivax malaria relapse	Clinical infectious diseases : an official publication of the Infectious Diseases Society of America	39	8	1095-103	
Hamedi et. al.	2004	Therapeutic efficacy of artesunate in Plasmodium vivax malaria in Thailand	The Southeast Asian journal of tropical medicine and public health	35	3	570-4	
Leslie et. al.	2004	Compliance with 14-day primaquine therapy for radical cure of vivax malaria--a randomized placebo-controlled trial comparing unsupervised with supervised	Transactions of the Royal Society of Tropical Medicine and Hygiene	98	3	168-73	
Vijaykadga et. al.	2004	Assessment of therapeutic efficacy of chloroquine for vivax malaria in Thailand	The Southeast Asian journal of tropical medicine and public health	35	3	566-9	Primaquine given at end of follow up
Dunne et. al.	2005	A double-blind, randomized study of azithromycin compared to chloroquine for the treatment of Plasmodium vivax malaria in India	The American journal of tropical medicine and hygiene	73	6	1108-11	
Yeramian et. al.	2005	Efficacy of DB289 in Thai patients with Plasmodium vivax or acute, uncomplicated Plasmodium falciparum infections	The Journal of infectious diseases	192	2	319-22	
Alvarez et. al.	2006	Efficacy of three chloroquine-primaquine regimens for treatment of Plasmodium vivax malaria in Colombia	The American journal of tropical medicine and hygiene	75	4	605-9	
Carmona-Fonseca et. al.	2006	Plasmodium vivax malaria: treatment of primary attacks with primaquine, in three different doses, and a fixed dose of chloroquine, Antioquia, Colombia, 2003-2004	Biomedica : revista del Instituto Nacional de Salud	26	3	353-65	Details same as Ref No. 93
Haghdoost et. al.	2006	Estimating the relapse risk of Plasmodium vivax in Iran under national chemotherapy scheme using a novel method	Journal of vector borne diseases	43	4	168-72	
Krudsood et. al.	2006	Safety and tolerability of elubaquine (bulaquine, CDRI 80/53) for treatment of Plasmidium vivax malaria in Thailand	The Korean journal of parasitology	44	3	221-8	

Additional File 3: Study References

Authors	Year	Title	Journal	Volume	Issue	Pages	Reason for Exclusion
Maguire et. al.	2006	Mefloquine is highly efficacious against chloroquine-resistant Plasmodium vivax malaria and Plasmodium falciparum malaria in Papua, Indonesia	Clinical infectious diseases : an official publication of the Infectious Diseases Society of America	42	8	1067-72	
Tasanor et. al.	2006	Clinical-parasitological response and in-vitro sensitivity of Plasmodium vivax to chloroquine and quinine on the western border of Thailand	Transactions of the Royal Society of Tropical Medicine and Hygiene	100	5	410-8	
Dao et. al.	2007	Vivax malaria: preliminary observations following a shorter course of treatment with artesunate plus primaquine	Transactions of the Royal Society of Tropical Medicine and Hygiene	101	6	534-9	
Hasugian et. al.	2007	Dihydroartemisinin-piperaquine versus artesunate-amodiaquine: superior efficacy and posttreatment prophylaxis against multidrug-resistant Plasmodium falciparum and	Clinical infectious diseases : an official publication of the Infectious Diseases Society of America	44	8	1067-74	Primaquine given at end of follow up
Krudsood et. al.	2007	Clinical efficacy of chloroquine versus artemether-lumefantrine for Plasmodium vivax treatment in Thailand	The Korean journal of parasitology	45	2	111-4	
Elmes et. al.	2008	The efficacy and tolerability of three different regimens of tafenoquine versus primaquine for post-exposure prophylaxis of Plasmodium vivax malaria in the	Transactions of the Royal Society of Tropical Medicine and Hygiene	102	11	1095-101	Post Exposure Prophylaxis
Krudsood et. al.	2008	High-dose primaquine regimens against relapse of Plasmodium vivax malaria	The American journal of tropical medicine and hygiene	78	5	736-40	
Leslie et. al.	2008	A randomised trial of an eight-week, once weekly primaquine regimen to prevent relapse of plasmodium vivax in Northwest Frontier Province, Pakistan	PloS one	3	8	e2861	
Carmona-Fonseca et. al.	2009	Prevention of Plasmodium vivax malaria recurrence: efficacy of the standard total dose of primaquine administered over 3 days	Acta tropica	112	2	188-92	
Lee et. al.	2009	Biological resistance of hydroxychloroquine for Plasmodium vivax malaria in the Republic of Korea	The American journal of tropical medicine and hygiene	81	4	600-4	
Moon et. al.	2009	Recurrence rate of vivax malaria in the Republic of Korea	Transactions of the Royal Society of Tropical Medicine and Hygiene	103	12	1245-9	
Orjuela-Sanchez et. al.	2009	Recurrent parasitemias and population dynamics of Plasmodium vivax polymorphisms in rural Amazonia	The American journal of tropical medicine and hygiene	81	6	961-8	
Carmona-Fonseca et. al.	2010	Vivax malaria in children: Recurrences with standard total dose of primaquine administered in 3 vs. 7 days	IATREIA	23	1	10--20	No translation available
Pukrittayakamee et. al.	2010	A comparison of two short-course primaquine regimens for the treatment and radical cure of Plasmodium vivax malaria in Thailand	The American journal of tropical medicine and hygiene	82	4	542-7	
Takeuchi et. al.	2010	Directly-observed therapy (DOT) for the radical 14-day primaquine treatment of Plasmodium vivax malaria on the Thai-Myanmar border	Malaria journal	9		308	
Yeshiwondim et. al.	2010	Therapeutic efficacy of chloroquine and chloroquine plus primaquine for the treatment of Plasmodium vivax in Ethiopia	Acta tropica	113	2	105-13	
Maneeboonyang et. al.	2011	Directly observed therapy with primaquine to reduce the recurrence rate of plasmodium vivax infection along the Thai-Myanmar border	The Southeast Asian journal of tropical medicine and public health	42	1	Sep-18	
Muhamed et. al.	2011	Monitoring of clinical efficacy and in vitro sensitivity of Plasmodium vivax to chloroquine in area along Thai Myanmar border during 2009-2010	Malar J	10		44	
Poravuth et. al.	2011	Pyronaridine-artesunate versus chloroquine in patients with acute Plasmodium vivax malaria: a randomized, double-blind, non-inferiority trial	PloS one	6	1	e14501	Primaquine given at end of follow up
