# Supplementary material for: Primaquine radical cure of Plasmodium vivax: a critical review of the literature
Source: Malar J. 2012 Aug 17;11:280. doi: 10.1186/1475-2875-11-280 (PMC3489597; doi:10.1186/1475-2875-11-280)
Supplement: Additional file 4 — Study design of articles included in the analysis. [file 1475-2875-11-280-S4.pdf]

Additional File 4: Design of Studies Included in the Analysis

First Author	Year of Publication	Country	No.of trial arms	Prospective Study	Patient Enrolment	Parasitemia Criteria	Randomization	Control Arm	Age Lower Limit (yrs)	Age Upper Limit (yrs)	Duration of Follow Up (days)
Di Lorenzo	1953	USA	2	Yes	Soldiers (Korean)	No	No	Yes			90
Alving	1953	USA	3	Yes	Soldiers (Korean)	No	No	Yes			120
Coatney	1953	USA	2	Yes	Soldiers (Korean)	No	No	No			180
Cooper	1953	USA	9	Yes	Volunteers (Chesson)	No	No	No	21	45	350
Thaeler	1953	Nicaragua	3	Yes	In hospital	No	No	No			360
Alving	1955	USA	3	Yes	Volunteers (Chesson)	No	No	No			365
Alving	1960	USA	8	Yes	Volunteers (Chesson)	No	No	Yes			120
Basavaraj	1960	India	1	No	Out patient	No	No	No			480
Mendoza	1963	Mexico	2	Yes	Community	No	No	No	1		270
Martelo	1969	USA	2	Yes	Soldiers (Vietnam)	No	No	No			180
Fisher	1970	USA	2	No	Soldiers (Vietnam)	No	No	No			180
Contacos	1973	USA	1	Yes	Volunteers (Chesson)	No	No	No			200
Sharma	1973	India	1	Yes	Community	No	No	No	1		365
Contacos	1974	USA	2	Yes	Volunteers (W Pakistan)	No	No	No			133
Miller	1974	USA	1	Yes	Volunteers (Chesson)	No	No	No			270
Kaplan	1974	USA	1	Yes	Soldiers (Somalia)	No	No	No			895
Saint-Yves	1977	PNG	3	Yes	Out patient	No	Yes	No	10	40	180
Clyde	1977	USA	1	Yes	Volunteers (Chesson)	No	No	No	23	41	293
Roy	1977	India	1	Yes	Community	No	No	No			365
Cedillos	1978	El Salvador	4	Yes	Community	No	No	No			270
Roy	1979	India	1	Yes	Community	No	No	No			365
Appavoo	1984	India	1	Yes	Out patient	No	No	No			365
Dixon	1985	Thailand	3	Yes	In hospital	<100,000/μL	No	Yes	18		28
Sinha	1989	India	1	Yes	Out patient	No	No	No	1		395
Singh	1990	India	2	Yes	Out patient	No	No	No			240
Prasad	1991	India	4	Yes	Community	No	No	No			1440
Bunnag	1994	Thailand	2	Yes	In hospital	No	Yes	No	15	60	540
Baird	1995	Indonesia	6	Yes	Community	>40/μL	Yes	Yes	6	54	28
Tan-ariya	1995	Thailand	1	Yes	Out patient	No	No	No	17	35	100
Jelinek	1995	Germany	1	Yes	Travelers	No	No	No	6	74	540
Srivastava	1996	India	3	Yes	Community	No	No	No	2		540

Additional File 4: Design of Studies Included in the Analysis

First Author	Year of Publication	Country	No.of trial arms	Prospective Study	Patient Enrolment	Parasitemia Criteria	Randomization	Control Arm	Age Lower Limit (yrs)	Age Upper Limit (yrs)	Duration of Follow Up (days)
Fryauff	1997	Indonesia	2	Yes	Community	0.001%-1%	No	No	15		28
Smoak	1997	USA	2	Yes	Soldiers (Somalia)	No	No	No	18	39	300
Gogtay	1998	India	1	Yes	Out patient	No	No	No			180
Wilairatana	1999	Thailand	4	Yes	In hospital	No	No	No	15	65	28
Luxemburger	1999	Thailand	2	Yes	Out patient	No	No	Yes	2	55	63
Gogtay	1999	India	3	Yes	In hospital	No	Yes	Yes	16	63	180
Li	1999	China	3	Yes	Out patient	Unknown	No	No	4	53	270
Rowland	1999	Pakistan	2	Yes	Out patient	No	Yes	Yes	3		300
Fang	1999	Taiwan	1	No	Travelers	No	No	No	6	64	540
Pukrittayakamee	2000	Thailand	9	Yes	In hospital	No	Yes	Yes	15	64	28
Singh	2000	India	1	Yes	In hospital	No	No	No	14	70	28
Villalobos-Salcedo	2000	Brazil	2	Yes	Community	>100/µL	Yes	No	12	40	90
Bergonzoli	2000	Nicaragua, Costa Rica	4	Yes	Community	No	Yes	No	15		180
Schwartz	2000	Israel	1	No	Travelers (Ethiopia)	No	No	No			365
Buchachart	2001	Thailand	1	Yes	In hospital	No	No	No	12	60	28
Duarte	2001	Brazil	1	Yes	Community	No	No	No	14	77	180
Abdon	2001	Brazil	3	Yes	Out patient	No	Yes	No	12	67	180
Adak	2001	India	3	Yes	Out patient	No	Yes	Yes	15	65	365
Dua	2001	India	1	Yes	Out patient	No	No	No			540
Lacy	2002	Indonesia	1	Yes	Community	No	No	No	15	48	28
Hamedi	2002	Iran	1	Yes	In hospital	2000-35,000/µl	No	No	15	59	28
Congpuong	2002	Thailand	1	Yes	In hospital	No	No	No	14		28
Yadav	2002	India	2	Yes	Community	No	No	Yes	1		365
Silachamroon	2003	Thailand	4	Yes	In hospital	No	Yes	No	15		28
Valibayov	2003	Azerbaijan	1	Yes	Out patient	>250/µL	No	No	6	85	28
Machado	2003	Brazil	1	Yes	Community	No	No	No			30
Fernandopulle	2003	Sri Lanka	1	Yes	In hospital	No	No	No			180
Rajgor	2003	India	2	Yes	Out patient	No	Yes	Yes	16	88	180
Da Silva	2003	Brazil	8	Yes	Out patient	No	Yes	No	14		180
Hamedi	2004	Thailand	1	Yes	In hospital	No	No	No	12	56	28
Walsh	2004	Thailand	5	Yes	In hospital	No	Yes	Yes	18	55	168

Additional File 4: Design of Studies Included in the Analysis

First Author	Year of Publication	Country	No.of trial arms	Prospective Study	Patient Enrolment	Parasitemia Criteria	Randomization	Control Arm	Age Lower Limit (yrs)	Age Upper Limit (yrs)	Duration of Follow Up (days)
Leslie	2004	Pakistan	3	Yes	Out patient	No	Yes	Yes	3		270
Dunne	2005	India	2	Yes	In hospital	<100,000/µL	Yes	No	18	65	28
Yeramian	2005	Thailand	1	Yes	In hospital	<25,000/µL	No	No	18	30	28
Maguire	2006	Indonesia	2	Yes	Community	No	Yes	No	6	58	28
Krudsood	2006	Thailand	2	Yes	In hospital	No	Yes	No	16	51	28
Tasanor	2006	Thailand	2	Yes	Out patient	1000-22,000/µl	Yes	No	15	60	28
Alvarez	2006	Columbia	3	Yes	Out patient	No	Yes	No	15		180
Haghdoost	2006	Iran	1	No	Out patient	No	No	No			2555
Dao	2007	Vietnam	1	Yes	In hospital	500-50,000/µL	No	No			28
Krudsood	2007	Thailand	2	Yes	In hospital	No	Yes	No	15	65	28
Krudsood	2008	Thailand	6	Yes	In hospital	No	Yes	No	12	60	28
Leslie	2008	Pakistan	3	Yes	Out patient	No	Yes	Yes	4	80	330
Lee	2009	Republic of Korea	1	Yes	In hospital	No	No	No	19	50	28
Carmona-Fonseca	2009	Columbia	4	Yes	Out patient	>1000/µL	Yes	No	2	50	120
Orjuela-Sanchez	2009	Brazil	2	Yes	Community	No	No	No	1	75	336
Moon	2009	Republic of Korea	1	No	In hospital	No	No	No			660
Pukrittayakamee	2010	Thailand	2	Yes	In hospital	No	Yes	No	14	61	28
Takeuchi	2010	Thailand	2	Yes	Community	No	Yes	No			90
Yeshiwondim	2010	Ethiopia	3	Yes	Out patient	No	Yes	Yes	4	65	157
Muhamed	2011	Thailand	1	Yes	Out patient	1000-100,000/µ	No	No	15	60	42
Maneeboonyang	2011	Thailand	2	Yes	Community	No	No	No	1	80	90
Pukrittayakamee	1994a	Thailand	3	Yes	In hospital	No	Yes	No	15	50	30
Pukrittayakamee	1994b	Thailand	3	Yes	In hospital	No	Yes	Yes	15	50	28
Looareesuwan	1999a	Thailand	2	Yes	In hospital	No	Yes	Yes	12	63	28
Looareesuwan	1999b	Thailand	1	Yes	In hospital	>40/µL	No	No	15	52	84
