# Supplementary material for: Primaquine radical cure of Plasmodium vivax: a critical review of the literature
Source: Malar J. 2012 Aug 17;11:280. doi: 10.1186/1475-2875-11-280 (PMC3489597; doi:10.1186/1475-2875-11-280)
Supplement: Additional file 5 — Recurrence rates reported for all primaquine treatment arms. [file 1475-2875-11-280-S5.pdf]

First Author	Year of Publication	Country	Partner drug	Duration of Treatment (days)	Total primaquine dose (mg/kg)	Primaquine Supervision	Duration of Follow Up (days)	Sample Size	Recurrence Rate (%) of P. vivax at end of study
**Very Low Dose Primaquine (≤ 2.5 mg/kg Total Dose)**									
Baird	1995	Indonesia	Chloroquine	3	2.50	Not stated	28	19	15.8
Dixon	1985	Thailand	Chloroquine	5	1.25	Not stated	28	40	0.0
Krudsood	2008	Thailand	Artesunate	5	2.50	Not stated	28	60	15.0
Di Lorenzo	1953	USA (Korea)	Chloroquine	7	1.75	Not stated	90	31	3.0
Villalobos-Salcedo	2000	Brazil	Chloroquine	5	2.50	All doses	90	30	20.0
Alving	1960	USA (Chesson)	Chloroquine	28	2.00	Not stated	120	41	90.0
Carmona-Fonseca	2009	Columbia	Chloroquine	3	1.75	All doses	120	26	50.0
Carmona-Fonseca	2009	Columbia	Chloroquine	3	2.50	All doses	120	26	57.7
Abdon	2001	Brazil	Chloroquine	5	2.50	All doses	180	40	20.0
Alvarez	2006	Columbia	Chloroquine	7	1.75	All doses	180	62	41.9
Alvarez	2006	Columbia	Chloroquine	3	0.75	All doses	180	65	49.2
Bergonzoli	2000	Nicaragua, Costa Rica	Chloroquine	5	2.00	All doses	180	30	0.0
Bergonzoli	2000	Nicaragua, Costa Rica	Chloroquine	1	0.75	All doses	180	25	4.0
Da Silva	2003	Brazil	Chloroquine	5	2.50	Not stated	180	26	15.4
Da Silva	2003	Brazil	Artesunate (100mg)	5	2.50	Not stated	180	25	16.0
Da Silva	2003	Brazil	Artesunate (200mg)	5	2.50	Not stated	180	20	25.0
Da Silva	2003	Brazil	Artesunate (150mg)	5	2.50	Not stated	180	20	25.0
Fernandopulle	2003	Sri Lanka	Not stated	5	1.25	All doses	180	6	83.3
Gogtay	1998	India	Chloroquine	5	1.25	All doses	180	100	15.0
Gogtay	1999	India	Chloroquine	5	1.25	All doses	180	62	25.8
Saint-Yves	1977	PNG	Chloroquine	1	0.75	All doses	180	9	67.0
Contacos	1973	USA (W Pakistan)	Chloroquine	5	1.25	Not stated	200	5	100.0
Singh	1990	India	Chloroquine	5	1.25	Not stated	240	995	13.2
Cedillos	1978	El Salvador	Amodiaquine	5	1.25	Part supervised	270	90	21.1
Cedillos	1978	El Salvador	Amodiaquine	1	0.75	Part supervised	270	67	23.9
Mendoza	1963	Mexico	Chloroquine	5	1.25	All doses	270	389	20.6
Rowland	1999	Pakistan	Chloroquine	5	1.25	All doses	300	250	51.2
Cooper	1953	USA (Chesson)	Quinine	14	2.33	Not stated	350	34	65.0
Cooper	1953	USA (Chesson)	Chloroquine	7	2.33	Not stated	350	10	80.0
Thaeler	1953	Nicaragua	Chloroquine	14	2.33	Not stated	360	121	0.0

First Author	Year of Publication	Country	Partner drug	Duration of Treatment (days)	Total primaquine dose (mg/kg)	Primaquine Supervision	Duration of Follow Up (days)	Sample Size	Recurrence Rate (%) of P. vivax at end of study
Adak	2001	India	Chloroquine	5	1.25	All doses	365	220	26.8
Appavoo	1984	India	Chloroquine	3	1.25	Not stated	365	425	3.8
Prasad	1991	India	Chloroquine	5	1.25	All doses	365	883	2.0
Roy	1977	India	Chloroquine	5	1.25	Not stated	365	6393	1.3
Roy	1979	India	Chloroquine	5	1.25	All doses	365	1389	0.7
Sharma	1973	India	Chloroquine	5	1.25	All doses	365	140	9.3
Yadav	2002	India	Chloroquine	5	1.25	Not stated	365	759	6.5
Sinha	1989	India	Chloroquine	5	1.25	All doses	395	725	6.9
Basavaraj	1960	India	Chloroquine	5	1.25	Not stated	480	563	6.0
Dua	2001	India	Chloroquine	5	1.25	Not stated	540	5541	9.2
Srivastava	1996	India	Chloroquine	5	1.25	Not stated	540	173	5.8
Prasad	1991	India	Chloroquine	5	1.25	All doses	720	1439	4.0
Prasad	1991	India	Chloroquine	5	1.25	All doses	1080	2484	5.7
Prasad	1991	India	Chloroquine	5	1.25	All doses	1440	8914	23.2
**Low Dose Primaquine (>2.5 to <5.0 mg/kg Total Dose)**									
Looareesuwan	1999a	Thailand	Chloroquine	14	3.5	All doses	28	441	0.5
Singh	2000	India	Chloroquine	14	3.5	Part supervised	28	75	16.0
Buchachart	2001	Thailand	Chloroquine	14	3.5	Not stated	28	364	0.0
Congpuong	2002	Thailand	Chloroquine	14	3.5	Not stated	28	26	0.0
Hamedi	2002	Iran	Chloroquine	14	3.5	All doses	28	39	0.0
Valibayov	2003	Azerbaijan	Chloroquine	14	3.5	Not stated	28	143	0.0
Hamedi	2004	Thailand	Artesunate	14	3.5	All doses	28	42	4.8
Yeramian	2005	Thailand	DB289	14	3.5	Not stated	28	9	11.1
Krudsood	2006	Thailand	Chloroquine	7	3.5	All doses	28	68	1.5
Maguire	2006	Indonesia	Mefloquine	14	3.5	All doses	28	310	1.3
Maguire	2006	Indonesia	Chloroquine	14	3.5	All doses	28	249	21.2
Tasanor	2006	Thailand	Chloroquine	14	3.5	All doses	28	24	0.0
Tasanor	2006	Thailand	Quinine	14	3.5	All doses	28	23	0.0
Krudsood	2007	Thailand	Chloroquine	14	3.5	Not stated	28	42	0.0
Krudsood	2007	Thailand	Artemether-Lumefantri	14	3.5	Not stated	28	38	2.6

Additional File 4: Recurrence Rates

First Author	Year of Publication	Country	Partner drug	Duration of Treatment (days)	Total primaquine dose (mg/kg)	Primaquine Supervision	Duration of Follow Up (days)	Sample Size	Recurrence Rate (%) of P. vivax at end of study
Krudsood	2008	Thailand	Artesunate	9	4.5	Not stated	28	56	4.0
Krudsood	2008	Thailand	Artesunate	7	3.5	Not stated	28	57	11.0
Lee	2009	Republic of Korea	Chloroquine	14	3.5	Part supervised	28	108	0.0
Pukrittayakamee	2010	Thailand	None	7	3.5	Not stated	28	31	30.0
Pukrittayakamee	1994b	Thailand	Chloroquine	14	3.5	Not stated	28	25	0.0
Pukrittayakamee	1994b	Thailand	None	14	3.5	Not stated	28	30	10.0
Pukrittayakamee	2000	Thailand	Chloroquine	14	3.5	Not stated	28	22	0.0
Pukrittayakamee	2000	Thailand	None	14	3.5	Not stated	28	26	11.5
Pukrittayakamee	1994a	Thailand	Chloroquine	14	3.5	Not stated	30	20	0.0
Pukrittayakamee	1994a	Thailand	Rifampicin	14	3.5	Not stated	30	20	5.0
Machado	2003	Brazil	Chloroquine	14	3.5	Not stated	30	30	0.0
Muhamed	2011	Thailand	Chloroquine	14	3.5	Part supervised	42	130	0.0
Luxemburger	1999	Thailand	Chloroquine	14	3.5	All doses	63	43	7.0
Villalobos-Salcedo	2000	Brazil	Chloroquine	14	3.5	All doses	90	31	6.5
Takeuchi	2010	Thailand	Chloroquine	14	3.5	All doses	90	90	3.3
Takeuchi	2010	Thailand	Chloroquine	14	3.5	Unsupervised	90	97	12.4
Maneeboonyang	2011	Thailand	Chloroquine	14	3.5	All doses	90	43	0.0
Maneeboonyang	2011	Thailand	Chloroquine	14	3.5	Unsupervised	90	33	15.1
Tan-ariya	1995	Thailand	Chloroquine	14	3.5	Part supervised	100	50	12.0
Alving	1953	USA (Korea)	Chloroquine	14	3.5	Not stated	120	348	0.0
Alving	1960	USA (Chesson)	Chloroquine	14	3.5	Not stated	120	60	27.0
Alving	1960	USA (Chesson)	Chloroquine	28	4.0	Not stated	120	20	30.0
Alving	1960	USA (Chesson)	Chloroquine	28	3.0	Not stated	120	15	40.0
Alving	1960	USA (Chesson)	Chloroquine	56	4.0	Not stated	120	61	55.7
Carmona-Fonseca	2009	Columbia	Chloroquine	14	3.5	All doses	120	66	15.2
Carmona-Fonseca	2009	Columbia	Chloroquine	3	3.5	All doses	120	63	58.7
Yeshiwondim	2010	Ethiopia	Chloroquine	14	3.5	All doses	157	132	3.0
Walsh	2004	Thailand	Chloroquine	14	3.5	All doses	168	12	25.0
Coatney	1953	USA (Korea)	None	14	3.5	Not stated	180	294	0.0
Martelo	1969	USA (Vietnam)	Chloroquine	14	3.5	Not stated	180	21	14.3
Fisher	1970	USA (Vietnam)	Chloroquine	14	3.5	All doses	180	133	7.5

Additional File 4: Recurrence Rates

First Author	Year of Publication	Country	Partner drug	Duration of Treatment (days)	Total primaquine dose (mg/kg)	Primaquine Supervision	Duration of Follow Up (days)	Sample Size	Recurrence Rate (%) of P. vivax at end of study
Gogtay	1999	India	Chloroquine	14	3.5	All doses	180	63	0.0
Bergonzoli	2000	Nicaragua, Costa Rica	Chloroquine	14	3.5	All doses	180	26	0.0
Bergonzoli	2000	Nicaragua, Costa Rica	Chloroquine	9	2.8	All doses	180	28	3.6
Abdon	2001	Brazil	Chloroquine	7	3.5	All doses	180	39	0.0
Abdon	2001	Brazil	Chloroquine	14	3.5	All doses	180	40	5.0
Duarte	2001	Brazil	Chloroquine	14	3.5	Part supervised	180	50	14.0
Da Silva	2003	Brazil	Artesunate (100mg)	7	3.5	Not stated	180	22	0.0
Da Silva	2003	Brazil	Chloroquine	7	3.5	Not stated	180	25	4.0
Da Silva	2003	Brazil	Artesunate (200mg)	7	3.5	Not stated	180	20	5.0
Da Silva	2003	Brazil	Artesunate (150mg)	7	3.5	Not stated	180	24	20.8
Rajgor	2003	India	Chloroquine	14	3.5	All doses	180	103	5.8
Alvarez	2006	Columbia	Chloroquine	15	3.5	All doses	180	64	18.8
Mendoza	1963	Mexico	Chloroquine	14	3.5	All doses	270	363	10.2
Miller	1974	USA (Chesson)	Chloroquine	14	3.5	Not stated	270	57	3.5
Li	1999	China	Chloroquine	8	3.0	All doses	270	35	22.9
Leslie	2004	Pakistan	Chloroquine	14	3.5	All doses	270	210	19.0
Leslie	2004	Pakistan	Chloroquine	14	3.5	All doses	270	173	19.7
Smoak	1997	USA (Somalia)	Chloroquine / Quinine	14	3.5	Part supervised	300	32	25.0
Smoak	1997	USA (Somalia)	Chloroquine / Quinine	14	3.5	Part supervised	300	28	64.2
Rowland	1999	Pakistan	Chloroquine	14	3.5	All doses	300	100	32.0
Orjuela-Sanchez	2009	Brazil	Chloroquine	7	3.5	Part supervised	336	87	71.5
Cooper	1953	USA (Chesson)	Quinine	14	4.7	Not stated	350	34	15.0
Cooper	1953	USA (Chesson)	Chloroquine	7	3.5	Not stated	350	10	90.0
Orjuela-Sanchez	2009	Brazil	Chloroquine	7	3.5	Part supervised	355	77	36.6
Thaeler	1953	Nicaragua	Chloroquine	14	3.5	Not stated	360	151	0.0
Thaeler	1953	Nicaragua	Chloroquine	14	4.7	Not stated	360	49	0.0
Alving	1955	USA (Chesson)	Quinine	14	3.5	Not stated	365	19	5.3
Alving	1955	USA (Chesson)	Chloroquine	14	3.5	Not stated	365	19	26.3
Alving	1955	USA (Chesson)	None	14	3.5	Not stated	365	19	78.9
Saint-Yves	1977	PNG	Chloroquine	14	3.50	All doses	365	10	0.0
Schwartz	2000	Israel	Chloroquine	14	3.5	Not stated	365	15	33.3

First Author	Year of Publication	Country	Partner drug	Duration of Treatment (days)	Total primaquine dose (mg/kg)	Primaquine Supervision	Duration of Follow Up (days)	Sample Size	Recurrence Rate (%) of P. vivax at end of study
Bunnag	1994	Thailand	Chloroquine	14	3.5	Not stated	540	81	8.6
Jelinek	1995	Germany	Chloroquine	14	3.5	Not stated	540	56	12.5
Fang	1999	Taiwan	Chloroquine	14	3.5	Not stated	540	12	8.3
Moon	2009	Republic of Korea	Chloroquine	14	3.5	Not stated	660	3881	1.6
Haghdoost	2006	Iran	Chloroquine	14	3.5	Not stated	2555	12337	7.4
**High Dose Primaquine (≥ 5.0 mg/kg Total Dose)**									
Baird	1995	Indonesia	Chloroquine	27	10.0	Not stated	28	26	15.4
Baird	1995	Indonesia	Chloroquine (10mg/kg)	27	10.0	Not stated	28	23	13.0
Fryauff	1997	Indonesia	Halofantrine	28	10.0	All doses	28	26	0.0
Fryauff	1997	Indonesia	Chloroquine	28	10.0	All doses	28	27	11.1
Wilairatana	1999	Thailand	Artesunate	14	7.0	Not stated	28	15	0.0
Wilairatana	1999	Thailand	Sulfadoxine-Pyrimethar	14	7.0	Not stated	28	19	0.0
Wilairatana	1999	Thailand	None	14	7.0	Not stated	28	17	0.0
Lacy	2002	Indonesia	Atovaquone/Proguanil	14	7.0	Not stated	28	16	0.0
Silachamroon	2003	Thailand	Artesunate (5d)	14	7.0	Not stated	28	142	0.0
Silachamroon	2003	Thailand	Artesunate (7d)	14	7.0	Not stated	28	157	0.0
Dao	2007	Vietnam	Artesunate	7	5.3	All doses	28	28	3.6
Krudsood	2008	Thailand	Artesunate	11	5.5	Not stated	28	48	0.0
Krudsood	2008	Thailand	Artesunate	14	7.0	Not stated	28	52	0.0
Krudsood	2008	Thailand	Artesunate	7	7.0	Not stated	28	49	4.0
Pukrittayakamee	2010	Thailand	None	7	7.0	Not stated	28	33	6.0
Looareesuwan	1999b	Thailand	Atovaquone/Proguanil	14	7.0	Not stated	84	35	5.7
Alving	1960	USA (Chesson)	Chloroquine	56	8.0	Not stated	120	51	5.9
Alving	1960	USA (Chesson)	Chloroquine	56	6.0	Not stated	120	40	10.0
Alving	1960	USA (Chesson)	Chloroquine	98	7.0	Not stated	120	30	20.0
Contacos	1974	USA (Chesson)	Chloroquine	56	6.0	Not stated	133	10	0.0
Martelo	1969	USA (Vietnam)	Chloroquine	56	6.0	Not stated	180	21	28.6
Fisher	1970	USA (Vietnam)	Chloroquine	56	6.0	Unsupervised	180	94	22.3
Clyde	1977	USA (Chesson)	Chloroquine	7	7.0	All doses	293	11	0.0
Leslie	2008	Pakistan	Chloroquine	14	7.0	All doses	330	54	1.9

First Author	Year of Publication	Country	Partner drug	Duration of Treatment (days)	Total primaquine dose (mg/kg)	Primaquine Supervision	Duration of Follow Up (days)	Sample Size	Recurrence Rate (%) of P. vivax at end of study
Leslie	2008	Pakistan	Chloroquine	56	6.0	All doses	330	68	5.9
Saint-Yves	1977	PNG	Chloroquine	14	5.3	All doses	365	10	0.0
Bunnag	1994	Thailand	Chloroquine	14	5.3	Not stated	540	86	1.2
Kaplan	1974	USA (Somalia)	Chloroquine	21	5.3	All doses	895	207	4.3
**Primaquine Dose Not Stated**									
Dunne	2005	India	Azithromycin	14	Not Stated	Not stated	28	100	0.0
Dunne	2005	India	Chloroquine	14	Not Stated	Not stated	28	97	0.9
**Control Arms (No Primaquine)**									
Dixon	1985	Thailand	Chloroquine		No Primaquine		28	11	0.0
Pukrittayakamee	1994b	Thailand	Chloroquine		No Primaquine		28	30	0.0
Baird	1995	Indonesia	Chloroquine		No Primaquine		28	23	87.0
Baird	1995	Indonesia	Chloroquine		No Primaquine		28	22	68.2
Looareesuwan	1999a	Thailand	Chloroquine		No Primaquine		28	445	0.5
Pukrittayakamee	2000	Thailand	Chloroquine		No Primaquine		28	21	0.0
Walsh	2004	Thailand	Chloroquine		No Primaquine		56	10	80.0
Luxemburger	1999	Thailand	Chloroquine		No Primaquine		63	69	55.1
Di Lorenzo	1953	USA (Korea)	Chloroquine		No Primaquine		90	46	50.0
Alving	1953	USA (Korea)	Chloroquine		No Primaquine		120	355	38.6
Alving	1960	USA (Chesson)	Chloroquine		No Primaquine		120	74	95.9
Yeshiwondim	2010	Ethiopia	Chloroquine		No Primaquine		157	108	8.3
Gogtay	1999	India	Chloroquine		No Primaquine		180	60	11.7
Rajgor	2003	India	Chloroquine		No Primaquine		180	101	12.9
Leslie	2004	Pakistan	Chloroquine		No Primaquine		270	212	40.6
Rowland	1999	Pakistan	Chloroquine		No Primaquine		300	250	51.6
Rowland	1999	Pakistan	Chloroquine		No Primaquine		300	100	49.0
Leslie	2008	Pakistan	Chloroquine		No Primaquine		330	68	32.4
Adak	2001	India	Chloroquine		No Primaquine		365	224	40.2
Yadav	2002	India	Chloroquine		No Primaquine		365	723	8.6
