# Supplementary material for: Primaquine radical cure of Plasmodium vivax: a critical review of the literature
Source: Malar J. 2012 Aug 17;11:280. doi: 10.1186/1475-2875-11-280 (PMC3489597; doi:10.1186/1475-2875-11-280)
Supplement: Additional file 9 — Severe adverse events reported. [file 1475-2875-11-280-S9.pdf]

Additional File 9: Severe Adverse Events

First Author	Year of Publication	Sample Size	Co-administered Drug	Primaquine Supervision	Duration of Treatment (days)	Total Primaquine Dose (mg/kg)	Day of Adverse Event	Adverse Event Reported	No. of Patients	Action Taken
Cooper	1953	34	Quinine	Not stated	14	4.67	Not Stated	Cyanosis	2	None
Fisher	1970	133	Chloroquine	All doses	14	3.5	Not Stated	Haemolysis (G6PDd)	1	None
Clyde	1977	11	Chloroquine	All doses	7	7	4	Severe abdominal cramps, vomiting and cyanosis	1	Treatment stopped
Silachamroon	2003	157	Artesunate	Not stated	14	7	4	13% reduction in haematocrit (G6PDd)	1	Treatment stopped
Silachamroon	2003	157	Artesunate	Not stated	14	7	6	14% and 8% reduction in haematocrit (G6PDd)	2	Treatment stopped
Silachamroon	2003	157	Artesunate	Not stated	14	7	7	16% reduction in haematocrit (G6PDd)	1	Treatment stopped
Dunne	2005	97	Chloroquine	Not stated	14	Not Stated	Not Stated	Maculopapular rash	1	Treatment stopped
Dunne	2005	97	Chloroquine	Not stated	14	Not Stated	Not Stated	Severe pruritis	1	Treatment stopped
