# Supplementary material for: Primaquine radical cure of Plasmodium vivax: a critical review of the literature
Source: Malar J. 2012 Aug 17;11:280. doi: 10.1186/1475-2875-11-280 (PMC3489597; doi:10.1186/1475-2875-11-280)
Supplement: Additional file 10 — Main findings of the literature review. [file 1475-2875-11-280-S10.pdf]

- There is marked heterogeneity in the design of studies assessing relapse prevention (radical cure) by primaquine in vivax malaria.

- The risk of recurrent malaria varies considerably according to primaquine dosing, partner drug, duration of follow up and the geographic location of the study.

- As relapse may occur many weeks after the initial infection if a slowly eliminated antimalarial is given in treatment, studies of relapse prevention should have long follow-up (≥8 weeks).

- Recurrence rates following treatment with very low dose primaquine (total dose ≤2.5 mg/kg) show no significant benefit compared to control patients receiving no anti-relapse medication.

- Low dose regimens (total dose >2.5mg/kg and <5.0 mg/kg) resulted in an adequate response (<10% recurrent infections) in 32% (26/82) of studies and were only better than a control arm in 50% (6/12) of studies.

- High dose primaquine regimens (total dose ≥ 5.0 mg/kg) have high efficacy (OR=0.03 [95%CI: 0.01-0.13]) compared with patients not receiving primaquine. Few high dose primaquine studies followed patients for more than 2 months.

- High dose regimens administered intermittently over a prolonged period of time (more than 28 days) have significantly lower effectiveness than regimens administered over shorter time courses.

- Reports of serious adverse events were rare in G6PD normal patients, and of a similar magnitude to that reported following other antimalarials such as mefloquine.
