# Supplementary material for: Primaquine radical cure of Plasmodium vivax: a critical review of the literature
Source: Malar J. 2012 Aug 17;11:280. doi: 10.1186/1475-2875-11-280 (PMC3489597; doi:10.1186/1475-2875-11-280)
Supplement: Additional file 11 — Key components in the design of studies of relapse prevention by primaquine in vivax malaria. [file 1475-2875-11-280-S11.pdf]

**Essential**	**Combination of primaquine with a blood schizontocide known to have high efficacy in the study population,** to minimise the risk of recrudescent infections. Primaquine efficacy may vary with partner drug and this must also be confirmed (see Box 3).
	**Prolonged duration of follow-up** (12 months) – Since relapses can occur over a 12 months period it is important to conduct studies with long duration of follow-up.
	**Inclusion of a control arm** – The risk and timing of *P. vivax* relapse varies with the location of the study site, hence the efficacy, safety and risk-benefit of primaquine needs to be gauged where ethically acceptable by comparison with a control arm (given no antirelapse medication).
	**Assessment of adverse events with particular attention to haemolysis**. Primaquine can cause haemolysis particularly in patients with G6PD deficiency and varies with the study population.
	**Multicentre assessment.** The risk of relapse varies between *P. vivax* strains and the intensity of sporozoite inoculation. The risk of hemolysis is related to the G6PD deficiency genotypes prevalent . Both vary regionally. Risk and benefit should be assessed in a range of locations.
**Desirable**	**Retreatment and following patients through multiple recurrences** with same treatment regimen. The clinical consequences of relapsing infections and the benefits of their prevention require characterisation of the total number of relapses following an initial infection.
	**Quantification of haematological recovery**. Both malaria and primaquine result in haemolysis, and thus risk benefit needs to be confirmed through close monitoring of haematological recovery.
