# Supplementary material for: Primaquine radical cure of Plasmodium vivax: a critical review of the literature
Source: Malar J. 2012 Aug 17;11:280. doi: 10.1186/1475-2875-11-280 (PMC3489597; doi:10.1186/1475-2875-11-280)
Supplement: Additional file 12 — Clinical research priorities for optimising the radical cure of P. vivax. [file 1475-2875-11-280-S12.pdf]

- Characterisation of true relapse rates in different geographic areas in the age group mainly affected.
- Assessment of efficacy and safety of short course, high dose primaquine treatment regimens.
- Development of robust point-of-care G6PD diagnostics.
- Investigation of the safety and efficacy of tafenoquine, a long acting 8 aminoquinoline.
- Defining the pharmacokinetic profile of primaquine in at risk populations.
- Confirmation of primaquine efficacy when combined with different blood schizontocidal partner drugs.
- Confirmation of effectiveness of primaquine regimens in clinical practice.
- Quantification of risk of clinically significant haemolysis according to primaquine dose and host susceptibility.
- Development of techniques to distinguish between relapse, reinfection and recrudescent infections.
